# MSLN-mediated activation of EGFR-ERK1/2 signaling drives liver metastasis in breast cancer

**DOI:** 10.1038/s41420-025-02835-9

**Published:** 2026-01-09

**Authors:** Jing Chen, Zexiu Lu, Guowu Zhang, Die Meng, Chao Chang, Jian Chen, Boxuan Wang, Yanran Tong, Yuhang Hai, Ming Lei, Xingyu Yang, Yubi Gan, Chaoqun Deng, Peijin Dai, Manran Liu, Xi Tang

**Affiliations:** 1 Department of Laboratory Medicine, The First Affiliated Hospital of Chongqing Medical University, Chongqing, China; 2 Key Laboratory of Laboratory Medical Diagnostics, Chinese Ministry of Education, Chongqing Medical University, Chongqing, China; 3 Department of Breast Surgery, The Affiliated Yongchuan Hospital of Chongqing Medical University, Chongqing, China; 4 Department of Breast and Thyroid Surgery, The First Affiliated Hospital of Chongqing Medical University, Chongqing, China

**Keywords:** Breast cancer, Cell growth

## Abstract

Breast cancer (BC) is the most prevalent malignant disease affecting female patients globally, with triple-negative breast cancer (TNBC) being the subtype linked to the poorest clinical outcome. The liver is a frequent metastatic site of breast cancer. Therefore, elucidating the mechanism underlying liver metastasis in TNBC is crucial for identifying effective diagnostic and therapeutic targets, which holds significant potential for guiding clinical treatment. This study aimed to identify key genes driving breast cancer liver metastasis and to explore their functional mechanisms. Using RNA sequencing of metastatic 4T1-HM3 and primary 4T1-Pri tumor cells, mesothelin (MSLN) was identified as significantly upregulated in metastatic TNBC cells and tissues, as confirmed by qRT-PCR, Western blot, and immunohistochemistry. Further investigations revealed that MSLN overexpression is strongly correlated with liver metastasis compared to metastases at other sites. Mechanistically, MSLN binds to epidermal growth factor receptor (EGFR) and activates the EGFR-ERK1/2 signaling axis, thereby promoting TNBC cell survival and proliferation during metastasis. Importantly, targeting MSLN with a paclitaxel/carboplatin combination effectively inhibited liver metastasis of hepatotropic TNBC in a mouse model. Therefore, our study elucidates the role of the MSLN-mediated EGFR-ERK1/2 signaling pathway in TNBC liver metastasis and highlights potential targeted therapies for treating TNBC liver metastasis.

## Introduction

Breast cancer (BC) is the most frequently diagnosed cancer in women and ranks as the second leading cause of cancer-related deaths in women [1]. Triple-negative breast cancer (TNBC) is highly aggressive, lacks critical endocrine and targeted therapeutic options, and is associated with the poorest prognosis [2]. Moreover, TNBC exhibits a higher propensity for metastasizing to distant organs [3]. The liver is among the most common metastatic sites for BC [4]. Even after diagnosis and initial tumor treatment, 20–30% of BC patients develop metastasis [5]. Liver metastasis has been reported as a critical indicator of poor overall survival (OS) in BC patients [6]. Additionally, TNBC has the worst prognosis among BC patients with liver metastasis [6, 7]. However, the detail mechanisms driving liver metastasis in TNBC are poor understand.

The organ tropism of tumor cells observed in tumor metastasis is one of the main attention-grabbing issues in metastasis research. Breast cancer frequently spreads to brain, liver, lungs, bones and lymph nodes [8]. Among breast cancer subtypes, luminal cancer frequently metastasizes to bone, whereas TNBC preferentially spreads to visceral organs [9]. Genetic alterations and gene expression characteristics of organ metastasis are the causes of organotropic metastasis of breast cancer. In general, the transcriptome profiles of metastases from different sites differ. For example, transforming growth factor β (TGF-β) promotes breast cancer metastasis to the lung, but it is irrelevant for bone metastasis, which depends on Src**-**mediated signaling pathways [10, 11]. Similarly, bone metastatic cancer cells display higher levels of IGF1 receptor (IGF1R) phosphorylation compared to brain metastatic cancer cells under insulin-like growth factor 1 (IGF1) stimulation [12]. However, the organ-specific metastasis-driving mechanisms underlying solid tumors keep in understanding.

Mesothelin (MSLN) is a glycosylphosphatidylinositol (GPI)-anchored protein localized on the cell surface [13, 14]. MSLN has restricted expression in normal tissues but is abnormally overexpressed in a variety of solid tumors including TNBC, and potentially play roles in tumor cell proliferation, survival [15]. This may be attributed to cellular signaling pathways associated with MSLN expression. For instance, MSLN was reported to upregulate cell cycle protein E through signal transduction and transcription factor activator protein 3 (STAT3), leading to increased proliferation and accelerated cell cycle progression in pancreatic cancer cells [16]. And MSLN overexpression could constitutively activate NF-κB to promote cell survival [17, 18]. Furthermore, MSLN was found to mediate intercellular protein interactions, such as MSLN binding with CA125, to promote ovarian tumor cell invasion and primary tumor progression [19]. However, whether MSLN acts a role for tumor’s distant organ metastasis remains unclear.

This study is the first to reveal the unique upregulation of MSLN in liver-metastatic TNBC cells and underscores its critical significance in TNBC liver metastasis. Furthermore, we found that MSLN protein in TNBC cells directly binds to EGFR, activating the EGFR-ERK1/2 signaling pathway, thereby enhancing the proliferation and viability of disseminated TNBC cells to facilitate liver metastases formation.

## Results

### MSLN is elevated in liver metastases of TNBC cells and correlates with TNBC prognosis

**Fig. 1:**
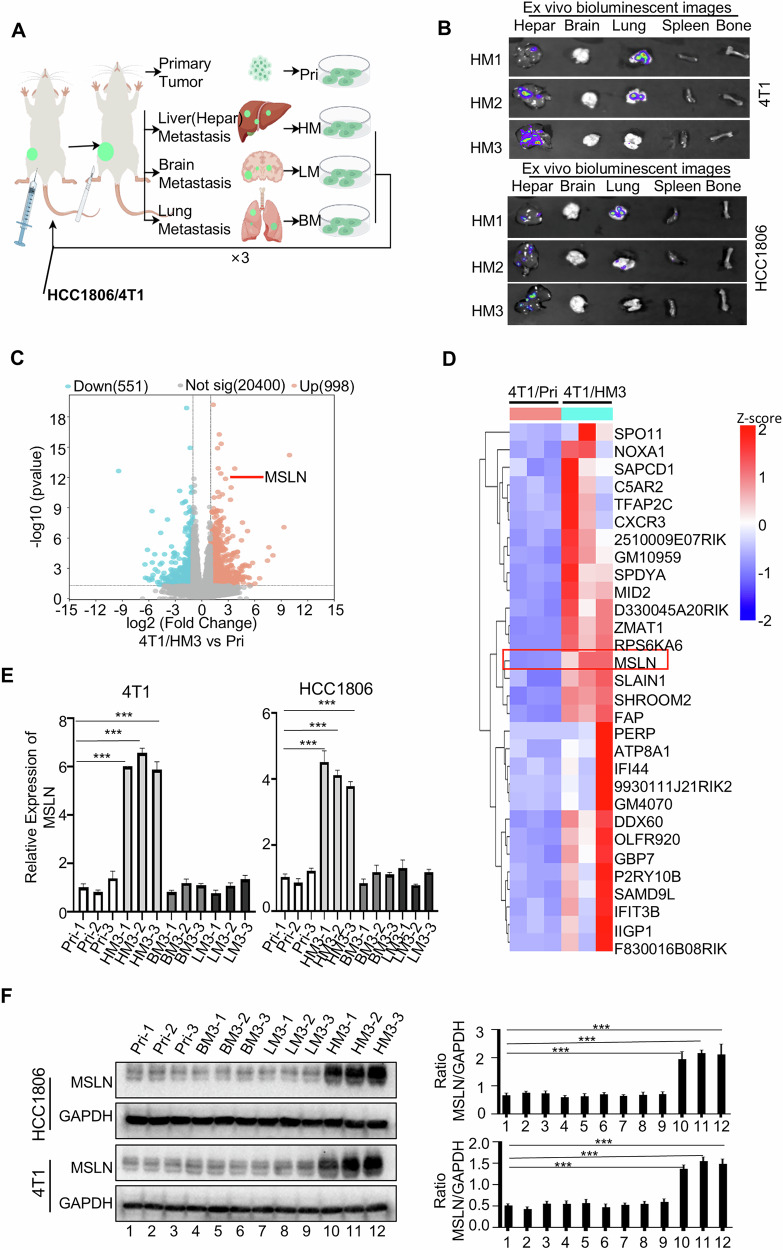
Elevated MSLN in liver metastatic TNBC cells. **A** Pattern diagram for the establishment of an organotropic metastasis mouse model: following in situ injection and systemic dissemination, organotropic metastatic variants were established through serial in vivo selection. Cells isolates from liver, brain, and lung metastases (first generation) were designated HM1, BM1, and LM1. Subsequent orthotopic re-implantation of these lines yielded second-generation derivatives (HM2, BM2, LM2), and the same procedure applied to the second-generation cells generated third-generation metastatic isolates (HM3, BM3, LM3). (“× 3” denotes three consecutive in vivo selections.) **B** Representative ex vivo bioluminescence images of HCC1806 and 4T1 cell hepatotropic metastasis in major organs. **C** Volcano plots showing gene expression differences (HM3: third-generation liver metastatic cells; Pri: Primary tumor cell), the RNAs that are markedly elevated and downregulated are indicated by red and blue dots, respectively. **D** Heatmap illustrating top 30 upregulated genes in 4T1/ HM3 compared to 4T1/Pri cells. **E** QRT-PCR to detect relative MSLN mRNA expression levels across metastatic organ (LM3/BM3: third-generation lung/brain metastatic cells). **F** WB analysis of MSLN protein levels in Pri and organ metastatic 4T1/HCC1806 cells. (*n* = 3 biological replicates, 3 samples of cells from 3 different mice) (Data are presented as the mean ± SD; ***P < 0.001).

To facilitate the study of organ metastasis of TNBC, organotropic metastatic models of TNBC were developed using 4T1 and HCC1806 cell lines via repeated fat pad injections and the selection and growth of metastatic clones in vivo (Fig. 1A). The obtained cell lines were named based on their source organs and injection cycles. Through ex vivo bioluminescence imaging (BLI) and metastatic nodule quantification, organotropic metastasis potential was validated in three derivative cells: HM3 (third-passage liver metastatic cells), BM3 (third-passage brain metastatic cell), and LM3 (third-passage lung metastatic cell) cell lines. These cells exhibited preferential metastasis to the liver, brain, and lungs, respectively (Fig. 1B, Fig. S1A–S1F) (The lung-tropic metastatic potential of 4T1/HM3 had been validated in our previous study [20]). Metastatic burden progressively increased across serial transplantations, as evidenced by escalating nodule counts in target organs (Fig. 1B, Fig. S1A–S1F). These data collectively establish a useful organ-preferential TNBC metastasis models. Furthermore, mice injected with the various organotropic metastatic derivatives of 4T1 were found to form more metastatic nodules than those injected with HCC1806 derivatives (Fig. S1D–S1F). This difference may be attributed to the murine origin of the 4T1 cell line, as syngeneic transplantation models are generally simpler to establish, and exhibit higher metastatic rates. To identify gene expression profiles linked to liver metastatic behavior, we conducted RNA sequencing analysis of 4T1/HM3 (third-passage liver metastatic cells) in comparison with their corresponding primary tumor cells (4T1/Pri). According to the volcano plots (Fig. 1C), 998 genes were upregulated and 551 were downregulated in 4T1-HM3 (fold change > 2.0 and *P* < 0.05). The top 30 upregulated genes were visualized in a heatmap; MSLN was significantly altered in the metastatic cells compared to their primary tumor cells (hereinafter referred to as Pri or Pri cells) (Fig. 1D, E, and Fig. S1G). Significantly, elevated MSLN expression was restricted to liver-metastatic cells, absent in brain or lung metastases (Fig. 1F). Furthermore, MSLN protein levels in liver-metastatic cells rose progressively with increased injections cycles (Fig. S1H). These results point to a possible function of MSLN in encouraging TNBC’s hepatotropic metastases.

**Fig. 2:**
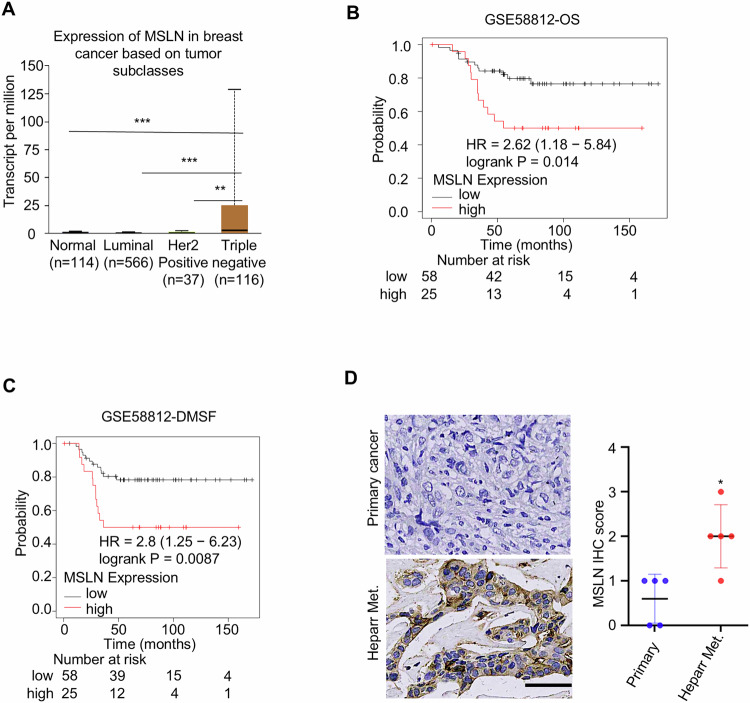
Elevated MSLN is associated with clinical features of TNBC. **A** Comparison of MSLN expression across human breast cancer subtypes using the UALCAN website. Kaplan–Meier plots illustrated overall survival (**B**) and distant metastasis-free survival (**C**) of TNBC patients with high or low MSLN expression. **D** MSLN expression in clinical primary of TNBC and liver metastases was showed in representative IHC images (Scale bars, 100 μm), and IHC score statistics were carried out (results represent as the mean ± SD). (*P < 0.05, **P < 0.01, ***P < 0.001).

To explore the clinical relevance of MSLN in liver metastasis of BC, we evaluated MSLN expression in the breast cancer TCGA database online using the UALCAN website and observed that MSLN exhibited higher expression level in TNBC compared to other BC subtypes (Fig. 2A). In addition, the enhanced MSLN had an inverse correlation with the overall survival (OS) and distant metastasis-free survival (DMFS) in TNBC patients (Fig. 2B, C). Importantly, there was an enhanced MSLN expression in TNBC liver metastases compared with primary tumor tissues (Fig. 2D). Then, we examined MSLN expression in other cancers associated with hepatotropic metastasis in several public datasets to broaden our findings. Notably, elevated MSLN expression was similarly detected in colorectal cancer (but not other tumor types) liver metastases (Fig. S2A–S2C), indicating that the dysregulation of MSLN in liver metastases may have cancer type limitations. Collectively, these findings demonstrate that the enhanced MSLN is linked to liver metastasis and poor prognosis in TNBC, with potential implications for metastatic targeting across gastrointestinal malignancies.

### MSLN promotes TNBC cell proliferation and survival to contribute liver metastasis of breast cancer

**Fig. 3:**
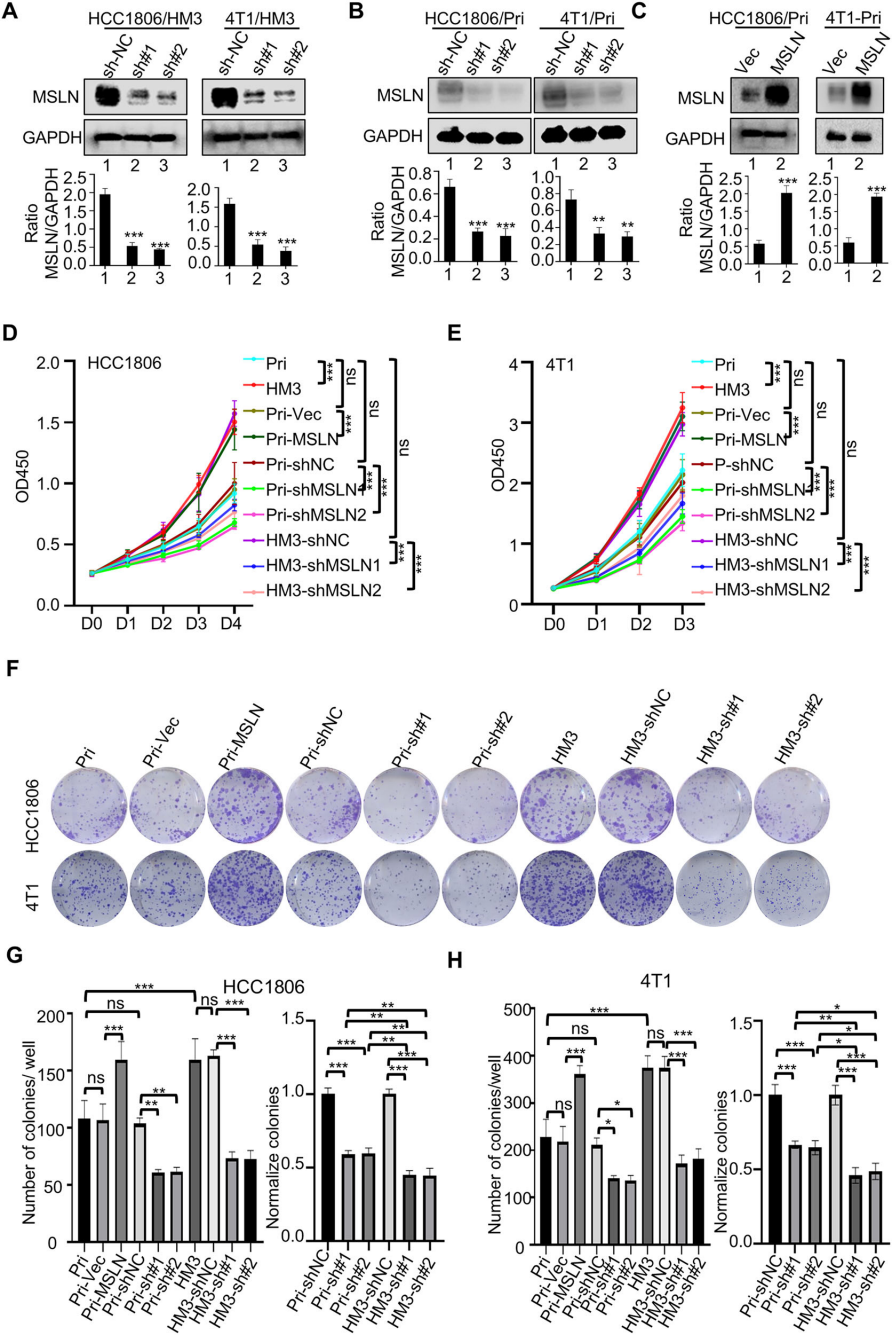
MSLN promotes TNBC proliferation and survival. Western blot analysis of MSLN knockdown efficiency in HM3 cells (**A**) and corresponding Pri cells (**B**) and MSLN proteins in ectopic MSLN overexpressing Pri cells (**C**) (*n* = 3). **D**, **E** CCK-8 was used to assess cell proliferation (*n* = 6). **F**–**H** Colony formation assays were determined to assess cell viability (*n* = 3). The left panel of **G**, **H** shows the clone number statistics for each group, and the right panel normalizes the number of clones in the MSLN knockdown group to their respective control group to compare the inhibitory effect of MSLN knockdown on the survival of Pri and HM3 cell lines. (*#, MSLN)* (Data are presented as the mean ± SD; ns no statistical difference, *P < 0.05, **P < 0.01, ***P < 0.001).

The metastatic cascade encompasses the invasion of cancer cells into tissues, their survival during circulation in blood, and subsequent colonization and proliferation in distant organs [21]. To investigate the potential function of MSLN in the process of liver metastasis, stable MSLN knockdown (shMSLN) was established in both high MSLN-expressing HM3 derivatives (4T1/HM3/shMSLN and HCC1806/HM3/shMSLN; 4T1/HM3/shNC and HCC1806/HM3/shNC as controls) and their relatively low MSLN-expressing Pri cells (4T1-Pri/shMSLN, HCC1806-Pri/shMSLN; 4T1-Pri/shNC and HCC1806-Pri/shNC works as controls) (Fig. 3A, B). Conversely, MSLN was overexpressed in primary cells (HCC1806-Pri/MSLN, 4T1-Pri/MSLN, and their Vec controls, HCC1806-Pri/Vec, 4T1-Pri/Vec) (Fig. 3C). Subsequent in vitro functional analyses unveiled that MSLN expression levels had no significant correlation with tumor cell invasion or migration capacity (Fig. S3A, B). Notably, ectopic MSLN overexpression in Pri cells significantly enhanced cellular proliferation (Fig. 3D, E) and viability (Fig. 3F–H) compared to their controls. This pro-proliferative effect mirrored the intrinsically elevated growth phenotype observed in high-MSLN-expressing HM3 derivatives versus Pri cells (Fig. 3D–H). Conversely, MSLN knockdown decreased proliferation and viability in both Pri and HM3 cells, with significantly amplified inhibitory effects in HM3 cells (Fig. 3D–H). This differential sensitivity is potentially attributable to HM3 cells’ elevated basal MSLN expression (Fig. 1F). Furthermore, TCGA data analysis revealed significant positive correlations between MSLN expression and proliferation markers *MKI67* (Ki-67) and *PCNA* in BC patients (Fig. S4A, B). To determine whether MSLN’s proliferative effect involved modulation of cell death, apoptosis analysis was performed. Neither MSLN knockdown nor overexpression significantly altered apoptosis rates (Fig. S4C–E), indicating that the observed effects on cell growth were primarily mediated through altered proliferation rather than regulation of cell death. Transient siRNA-mediated MSLN knockdown in HCC1806-Pri, 4T1-Pri, and their corresponding HM3 derivatives further validated these findings (Fig. S5A, B), that is, the effects on proliferation activity and apoptosis consistently mirrored those observed with stable knockdown (Fig. S5C–F). Collectively, these results suggest that MSLN plays a pivotal role in promoting the proliferation and survival of TNBC cells.

**Fig. 4:**
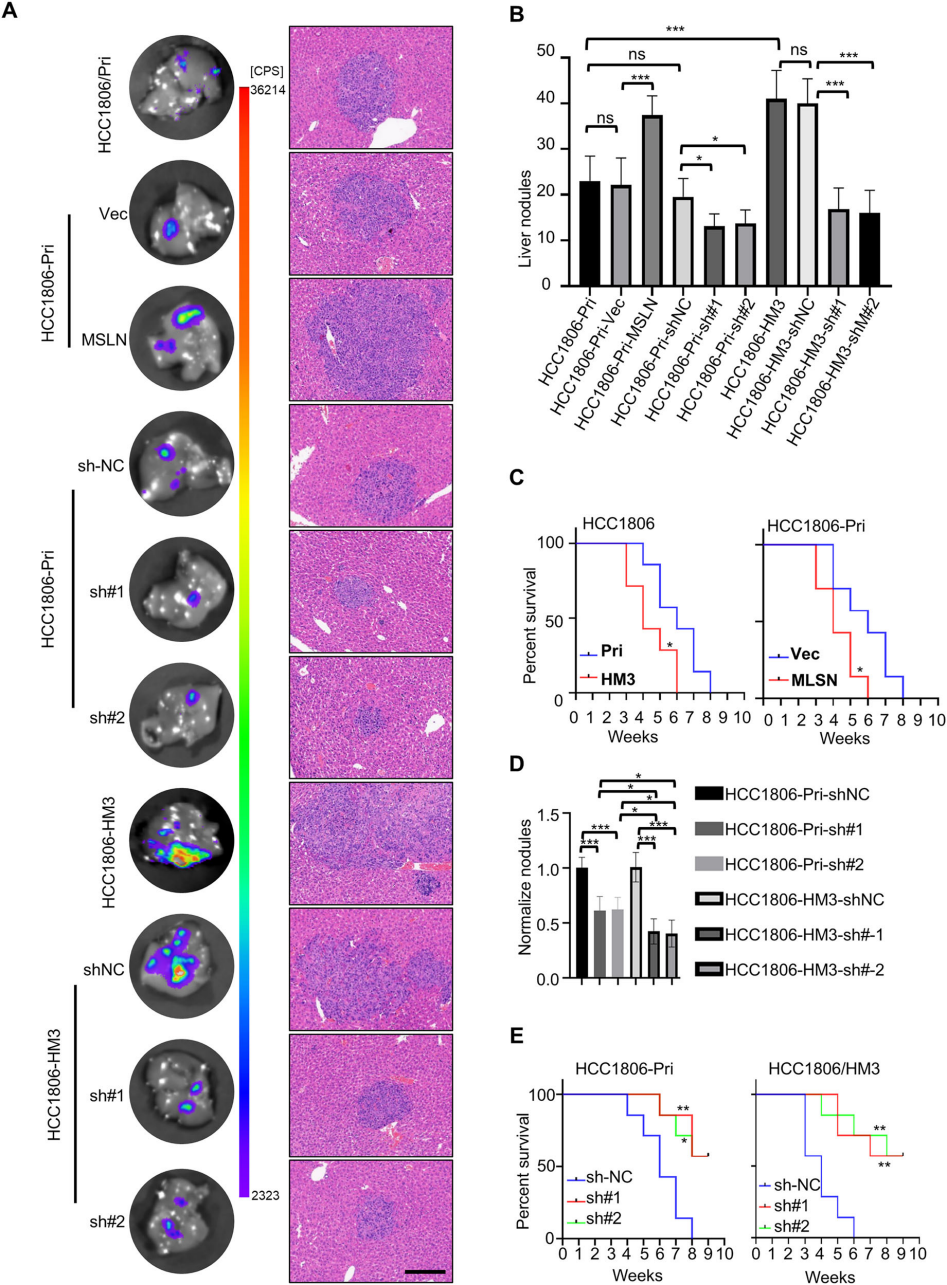
MSLN drives liver metastasis in TNBC. Mouse spleen injections with luc-indicator cells (HCC1806-derived cells), when mice were observed to be in a weak state, mice were given an intraperitoneally injected of D-luciferin substrate (150 mg/kg), yielded representative in vitro bioluminescence imaging (BLI) of liver and H&E images of liver metastases (**A**) (scale bar, 200 μm), liver metastatic nodule count statistics (**B**). **C** Kaplan–Meier Survival Curves of Indicated Cell-Injected Mice (*n* = 7). **D** The number of liver metastasis nodules in MSLN-knockdown groups was normalized to their respective control groups to compare the inhibitory efficacy of MSLN knockdown on metastasis in Pri and HM3 cell lines. **E** Kaplan–Meier Survival Curves of Indicated Cell-Injected Mice (*n* = 7). (*#, MSLN*) (Data are presented as the mean ± SD; ns no statistical difference, *P < 0.05, **P < 0.01, ***P < 0.001).

To determine whether MSLN has a causal role in hepatotropic metastasis, we examine the effects of MSLN in metastasis using a spleen-injected mouse model of liver metastasis. Consistent with in vitro observations, HM3 cells showed significantly enhanced liver metastatic capacity and concomitantly reduced mouse survival in comparison with their Pri cells, this phenotype could be recapitulated by MSLN overexpression in Pri cells (Fig. 4A–C, Fig. S6A–C). However, MSLN knockdown attenuated liver metastasis and prolonged survival in Pri cells, with amplified effects in high MSLN-expressing HM3 cells (Fig. 4A, B and D, E, Fig. S6A, B and D, E). These data demonstrate that MSLN functionally drives liver metastasis. To substantiate this finding, we overexpressed MSLN in wild-type human TNBC cells (BT549) and murine mammary carcinoma cells (E0771), both exhibiting intrinsically low MSLN expression (Fig. S7A, B). Ectopic MSLN overexpression augmented proliferation (Fig. S7C), survival (Fig. S7D), and liver metastatic activity in both systems (Fig. S7E–H). Thus, these data demonstrate that MSLN plays a critical role in the hepatic metastasis of TNBC cells.

### Transcription factor ELF1 upregulates MSLN expression in TNBC

**Fig. 5:**
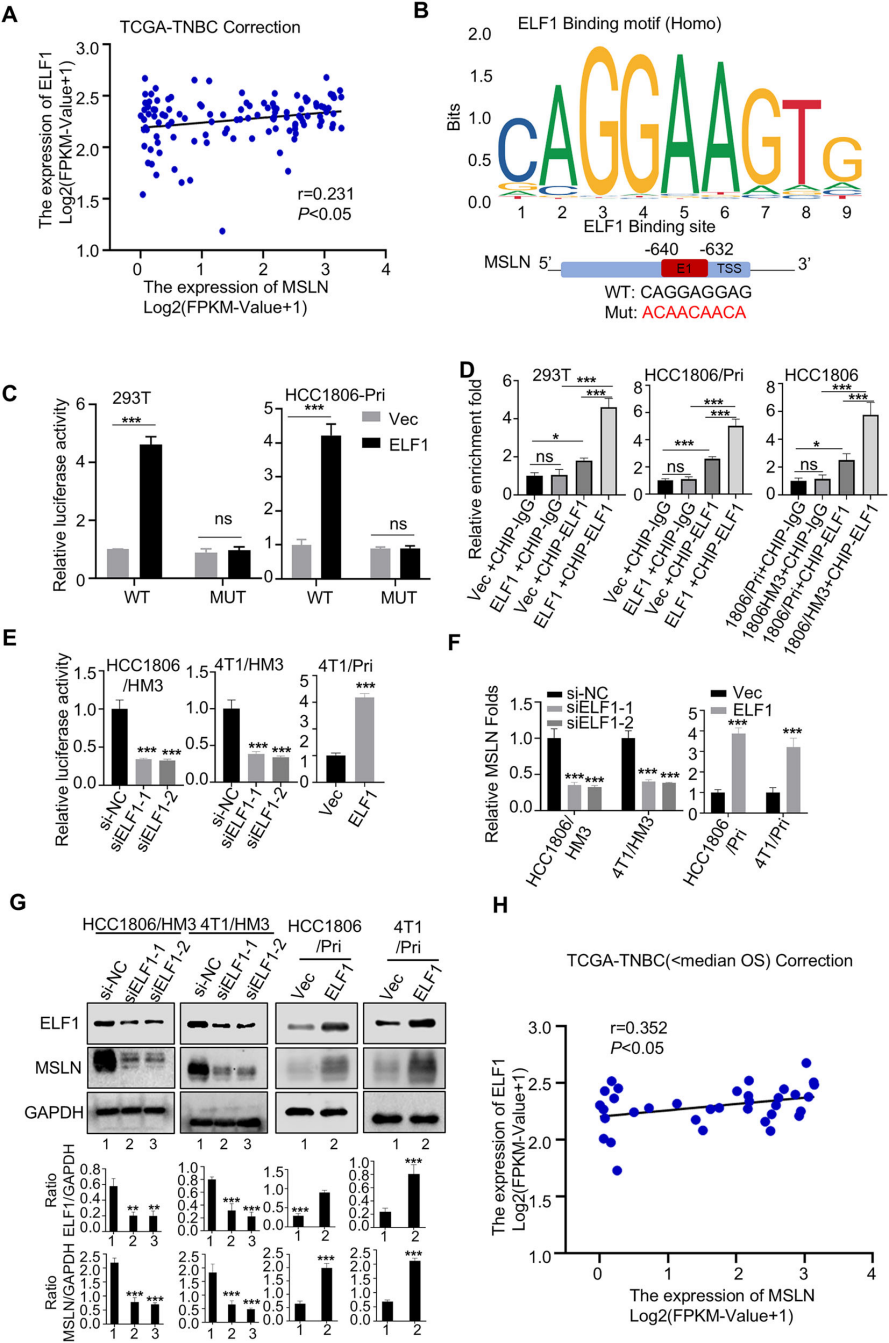
ELF1 positively regulates MSLN expression. **A** Pearson correlation analysis of the TCGA-TNBC dataset revealed a positive correlation between MSLN and ELF1 expression. **B** JASPAR database analysis identified the ELF1 recognition motif in the MSLN promoter (upper), and a schematic diagram (lower) illustrated potential ELF1-responsive elements (E1), and introduced an E1 sequence mutation (WT: CAGGAGGAG; Mut: ACAACAACA). **C** Luciferase reporter assay showed E1-dependent transcription regulation of MSLN by ELF1 in 293 T and HCC1806 Pri cells. **D** ChIP assays were performed to validate ELF1 binding to the MSLN promoter in 293 T and HCC1806-derived cells. ELF1 regulation of MSLN was analyzed via Luciferase reporter assay (**E**) qRT-PCR (**F**) and WB (**G**) in HCC1806 and 4T1-derived cells. **H** Pearson correlation analysis of ELF1 and MSLN expression in TNBC patients below median overall survival. (Data are presented as the mean ± SD; ns no statistical difference, *P < 0.05, ***P < 0.001).

To understand why MSLN is upregulated in hepatotropic metastases, we conducted bioinformatics analyses. Potential transcription factors (TFs) regulating the MSLN gene were identified through the PROMO database (http://alggen.lsi.upc.es/) and GTRD database (https://gtrd20-06.biouml.org/) (Fig. S8A). Correlation analysis between MSLN and these transcription factors was then conducted using breast cancer TCGA database (RNA-seq data), revealing that the expression of four transcription factors (ELF1, CEBPB, RELA, and IRF1) was positively correlated with MSLN, while AR expression was negatively correlated with MSLN (Table 1) (Fig. 5A). Checking their expression levels of these candidate transcription factors in the Pri and HM3 cells, ELF1 was significantly upregulated in the HM3 derivatives compared to the Pri cells (Fig. S8B–D).

**Table 1:** The correlation between MSLN and TFs, data from TCGA-TNBC database.

MSLN vs.	Pearson r	P value
ELF1	0.231	0.01
CEBPB	0.2432	0.009
RELA	0.2049	0.03
IRF1	0.195	0.04
AR	−0.3468	<0.001

We then used the JASPAR database to further identify the potential ELF1 binding sites (E1) in the MSLN promoter (Fig. 5B). To validate binding specificity, we engineered E1-disrupting mutations (Fig. 5B). Dual-luciferase reporter assays showed that ELF1 overexpression significantly enhanced MSLN promoter activity, whereas E1 mutations abolished this transactivation effect (Fig. 5C), establishing E1 as indispensable for ELF1-mediated transcriptional activation of MSLN. Chromatin immunoprecipitation (ChIP) assays further confirmed endogenous ELF1 binding to the MSLN promoter in 293 T (transfection-positive control) and HCC1806-P (TNBC model) cells (Fig. 5D). Notably, ELF1-overexpressing 293 T/HCC1806-P cells and intrinsically high-ELF1-expressing HM3 derivatives exhibited significantly enhanced binding versus controls (Fig. 5D). Functional validation revealed that ELF1 knockdown in HM3 cells reduced MSLN transcript activity and downregulated MSLN RNA/protein expression, while ELF1 overexpression in Pri cells reciprocally increased these parameters (Fig. 5E–G, and C. right panels), these data uncovered an ELF1-dependend MSLN upregulation in HM3 cells. To investigate whether ELF1 upregulates MSLN in different metastatic settings, we overexpressed ELF1 in BM3 and LM3 cells, neither of which showed baseline MSLN upregulation. Strikingly, ELF1 overexpression induced significant MSLN elevations in both BM3 and LM3 cells (Fig. S8E), suggesting its pan-metastatic regulatory potential. Clinically, we performed Pearson correlation analysis specifically within the TCGA-TNBC cohort exhibiting shorter overall survival (OS < median); and there was a significant positive correlation between ELF1 and MSLN expression (*r* = 0.352, *p* < 0.05; Fig. 5H). This correlation strength exceeded that observed in the entire TNBC cohort (*r* = 0.231, *p* < 0.05, Fig. 5A). These findings indicate that TNBC patients with high ELF1 expression and poor survival exhibit concomitant MSLN upregulation.

### MSLN interacts with EGFR and activates EGFR-ERK1/2 signaling

**Fig. 6:**
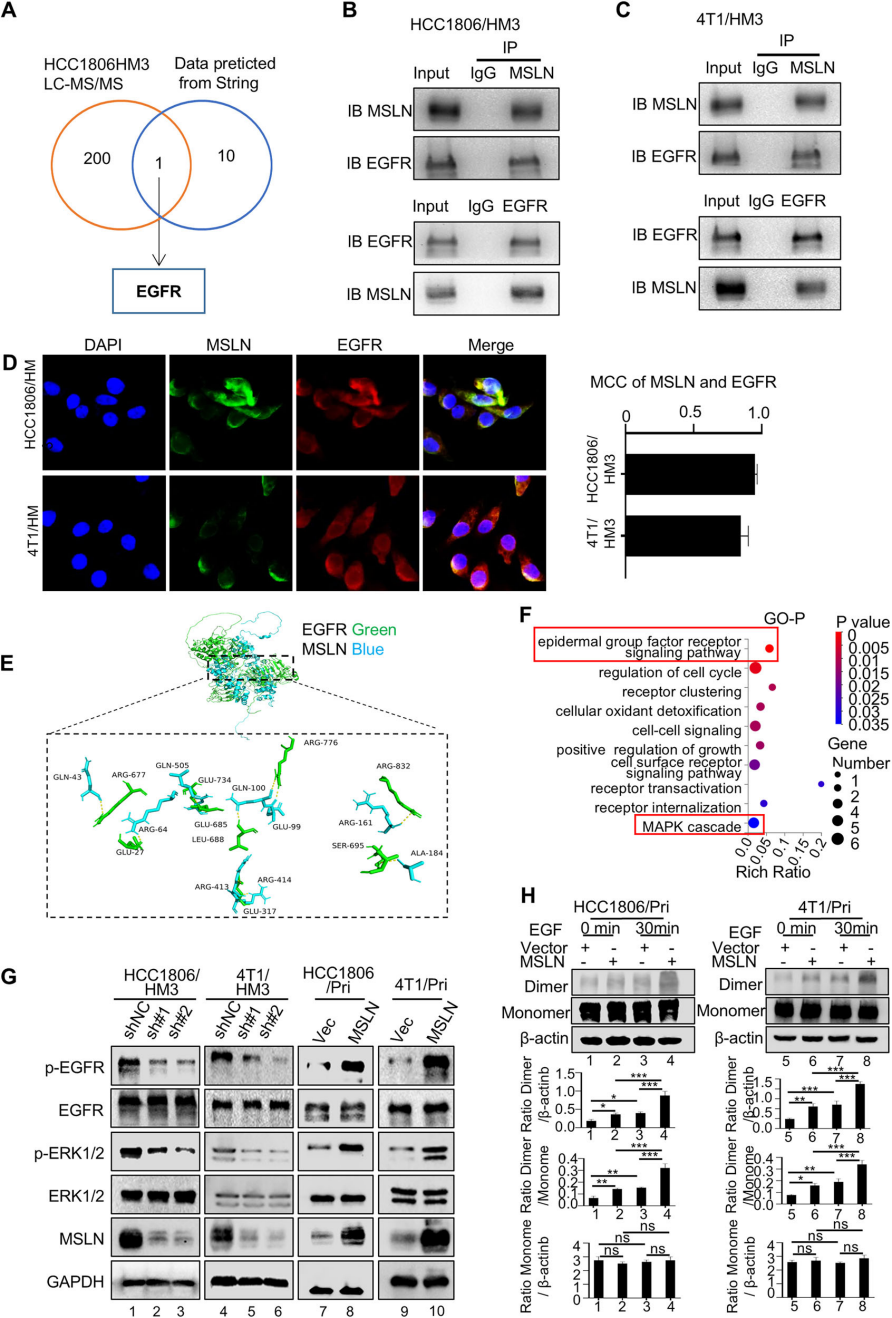
MSLN activates the EGFR-ERK1/2 signaling pathway. **A** Venn diagram illustrating potential interacting proteins of MSLN, including those identified by LC–MS/MS and those predicted through String databases. The direct interaction between MSLN and EGFR in HCC1806/HM3 (**B**) and 4T1/HM3 cells was verified by Co-IP assays (**C**) using anti-MSLN and anti-EGFR antibodies. **D** IF co-staining was performed to show the co-localization of MSLN (Green) and EGFR (Red) in HCC1806/HM3 and 4T1/HM3 cells. **E** Molecular docking demonstrates the best docking panel of MSLN (Blue) and EGFR (Green) proteins. **F** Significant GO clusters for the Biological Process categories enriched by the DEGs between MSLN-knockdown HCC1806/HM3 cells and control cells. **G** Western blotting to check the expression of the key factors of the EGFR-ERK1/2 signaling in cells with MSLN knockdown cells (HCC1806/HM3 and 4T1/HM3, left panels) and overexpression cells (HCC1806/Pri and 4T1/Pri, right panels) (*#, MSLN*). **H** Western blot analysis and quantification of EGFR dimerization levels in treated cells (*n* = 3). (Data are presented as the mean ± SD; ns no statistical difference, *P < 0.05, ** *P* < 0.01, ***P < 0.001).

MSLN is a GPI-anchored membrane protein, and previous studies have established a clear association between GPI-anchored and transmembrane proteins [22, 23]. In order to understand the relationship between MSLN and transmembrane proteins, we initially conducted a bioinformatics analysis using the STRING database to predict MSLN-interacting proteins, followed by affinity purification and Liquid Chromatography-Tandem mass spectrometry (LC-MS/MS) to isolate and identify the candidates of MSLN-interacting proteins in HCC1806/HM3 cells. EGFR was identified in the predicted bioinformatic data and the LC-MS/MS based experimental proteins (Fig. 6A). Further performing Co-immunoprecipitation assays, a physical interaction between MSLN and EGFR proteins was proved in HCC1806/HM3 cells (Fig. 6B), 4T1/HM3 cells (Fig. 6C) and the corresponding Pri cells (Fig. S9A). And MSLN and EGFR proteins were co-localized on the cell membranes of HCC1806/HM3 and 4T1/HM3 cells (Fig. 6D). Molecular docking analysis determined the optimal binding conformation between EGFR and MSLN proteins and revealed 11 potential binding sites at their interface (Fig. 6E, Supplementary Table 5), although these sites warrant rigorous experimental validation in future studies to confirm their biological relevance. These results indicate that MSLN protein interacts with EGFR.

To investigate the mechanism underlying MSLN-driven liver metastasis, we conducted RNA-Seq on MSLN-knockdown HM3 cells (HCC1806/HM3-shMSLN) and control cells (HCC1806/HM3-shNC) to identify differentially expressed genes (DEGs), followed by enrichment analysis. GO enrichment analysis-Biological Process (GO-P) (Fig. 6F) and KEGG enrichment analysis (Fig. S9B) suggest that EGFR signaling may be essential for MSLN to regulate biological functions. The activation of MSLN-mediated EGFR signaling was then verified through Western blotting experiments (WB) (Fig. 6G, Fig. S9C). Notably, immunoblot analysis revealed significantly enhanced EGFR activation in hepatotropic metastatic cells (HM3) compared to Pri cells, brain-tropic (BM3), and lung-tropic (LM3) derivatives, indicating organotropism-dependent EGFR hyperactivation in breast cancer liver metastases (Fig. S9D). Published studies have demonstrated that the EGFR signaling pathway activates the MAPK-ERK, PI3K-AKT, and JAK-STAT pathways [24]. To explore the specific downstream signaling of MSLN-mediated EGFR in HM3 cells, we conducted Western blot analysis and found that MSLN knockdown decreased ERK1/2 phosphorylation (Fig. 6G, Fig. S9C) without affecting PI3K/AKT (Fig. S9E and Fig. S10A) and JAK/STAT3 pathways (Fig. S10B, C), which were consistent with the GO and KEGG analysis results (Fig. 6F, and Fig. S9B). As established in prior literature, ligand binding (e.g. EGF) induces conformational changes in EGFR that promotes dimerization and activation of its intrinsic tyrosine kinase activity, culminating in trans-autophosphorylation and initiation of downstream signaling cascades [25]. Given our demonstration that MSLN activated the EGFR-ERK cascade, we hypothesized that MSLN may promote this critical dimerization step. Strikingly, cross-linking assays revealed that MSLN overexpression alone induced ligand-independent EGFR dimerization in the absence of EGF stimulation. Subsequent EGF treatment further potentiated dimer formation in MSLN-overexpressing cells (Fig. 6H). Collectively, these data demonstrate that upregulated MSLN in HM3 cells interacts with EGFR, promoting both ligand-independent and ligand-enhanced EGFR dimerization, thereby activating the EGFR-ERK1/2 signaling pathway.

### ELF1/MSLN/EGFR/ERK signaling axis plays an important role in hepatotropic metastasis of TNBC

**Fig. 7:**
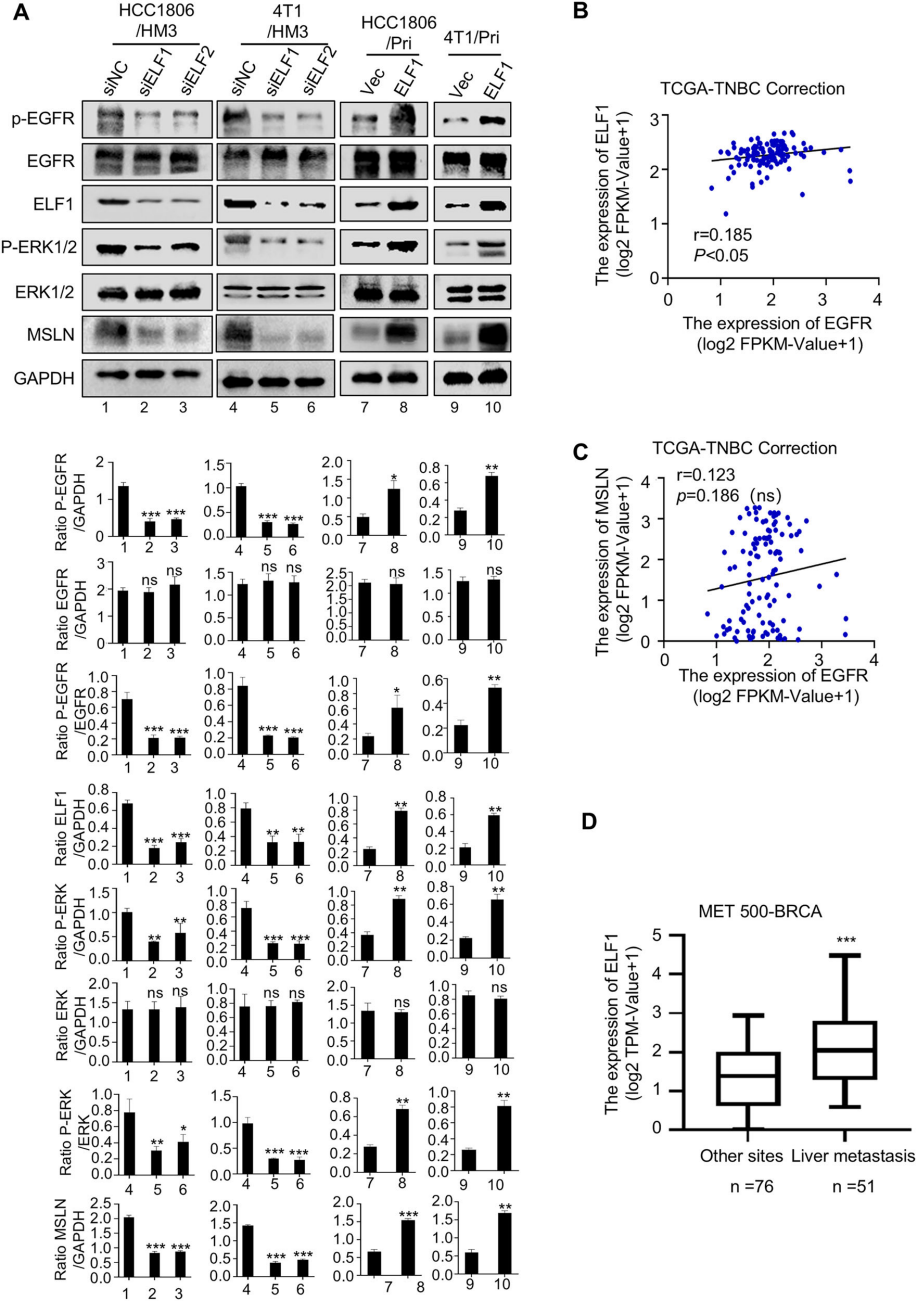
Regulatory effects of ELF1 on MSLN-EGFR-ERK1/2 signaling axis and clinical correlations. **A** Western blot analysis of key signaling molecules in the MSLN-EGFR-ERK1/2 axis following ELF1 knockdown or overexpression (*n* = 3). **B**, **C** Pearson correlation analysis of TCGA-TNBC data set showed non-significant correlation**s** between ELF1 -EGFR and MSLN-EGFR mRNA expression (*r* < 0.2). **D** Elevated ELF1 mRNA expression in liver metastases versus extrahepatic sites from MET500-BRCA RNA-seq data. (Data are presented as the mean ± SD, ns no statistical difference, *P < 0.05, **P < 0.01, ***P < 0.001).

To investigate the regulatory relationship between the transcription factor ELF1, MSLN, and the downstream EGFR signaling pathway, we manipulated ELF1 expression genetically, that is, knocked down endogenous ELF1 in HM3 cells or overexpression of ELF1 in their Pri counterparts (Fig. 7A). Notably, ELF1 depletion led to a significant downregulation of MSLN expression and a marked attenuation of EGFR-ERK1/2 signaling activation, whereas the total EGFR protein levels remained unchanged (Fig. 7A); conversely, ELF1 overexpression increased MSLN expression and increased phosphorylation of components in the EGFR-ERK1/2 pathway (Fig. 7A). These findings demonstrate that ELF1 transcriptionally upregulates MSLN, which in turn promotes EGFR activation through dimerization (Fig. 6H), rather than regulating EGFR expression itself. Consistent with this proposed mechanism, bioinformatics analyses of TCGA-TNBC cohorts revealed three key observations: (1) a positive correlation between ELF1 and MSLN expression (Fig. 5A); (2) no significant correlation between ELF1 and EGFR expression (Fig. 7B); and (3) no significant correlation between MSLN and EGFR expression (Fig. 7C). Notably, ELF1 was upregulated in BC liver metastases compared to other metastatic sites (Fig. 7D). Collectively, these results indicate that the ELF1/MSLN/EGFR/ERK signaling axis plays a critical role in orchestrating liver-tropic metastasis in TNBC.

### Targeting MSLN combined with Paclitaxel and Carboplatin enhances therapeutic efficacy in hepatotropic metastases

**Fig. 8:**
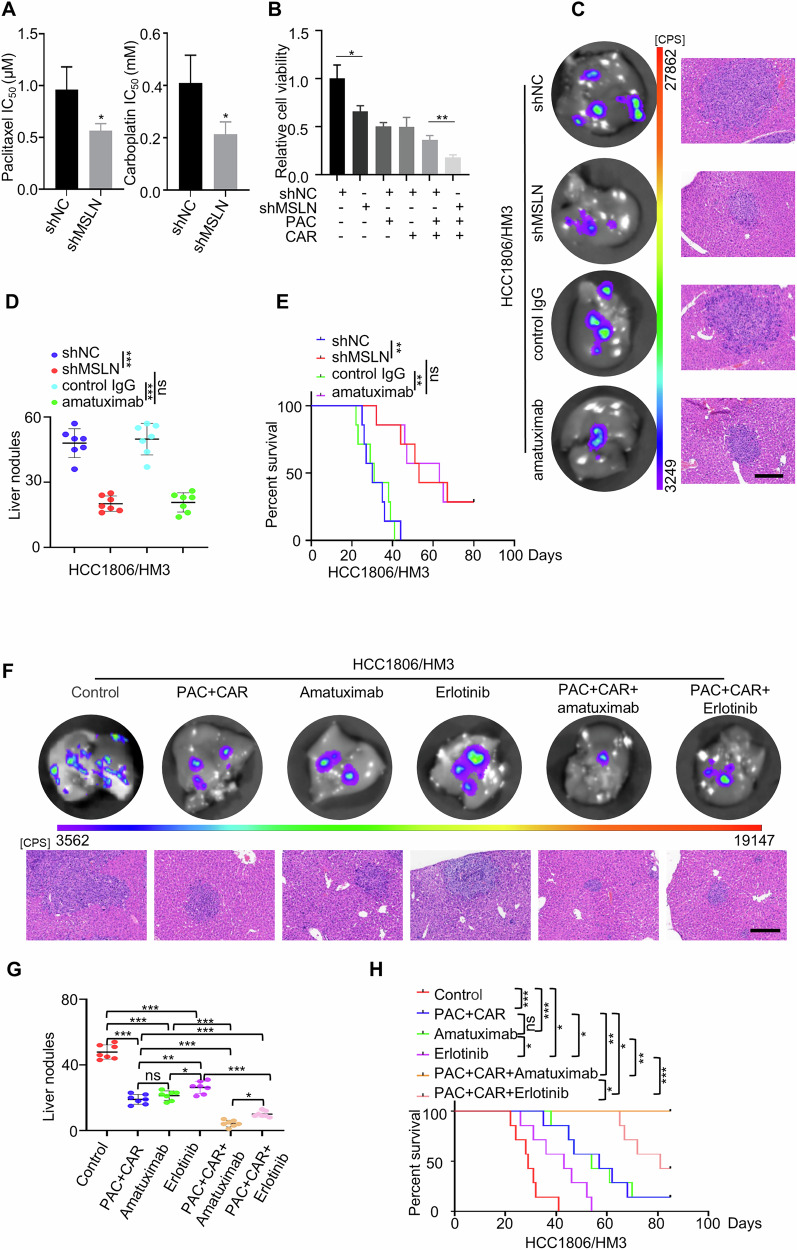
Combined therapy targeting MSLN with Chemotherapy in a hepatotropic metastasis mice model. **A**, **B** Hepatotropic metastatic cells (HCC1806/HM3) with either MSLN knockdown or control treatment were exposed to Paclitaxel (PAC) and Carboplatin (CAR) in serial dilutions. CCK8 assay to measure IC50 values (**A**) and cell viability (**B**) (*n* = 3). **C** Representative liver ex vivo BLI and H&E staining (scale bar: 200 μm) in mice treated with shMSLN genetic interventionor the MSLN inhibitor Amatuximab (60 mg/kg) (*n* = 7). Quantification of liver metastasis nodules (**D**) and the overall survival survival (**E**) of the above mice (*n* = 7). Representative liver ex vivo BLI and liver H&E staining (scale bar: 200 μm) (**F**) of mice with different treatment regimens, murine liver metastatic nodule statistics (**G**) and overall survival (**H**) (*n* = 7). (Data were presented as mean ± SD; ns no statistical difference, *P < 0.05, **P < 0.01, ***P < 0.001).

Previous studies have shown that Platinum combined with Paclitaxel is the preferred clinical approach to metastatic breast cancer; nevertheless, its efficacy remains limited [26]. We thus investigated whether MSLN knockdown could enhance the efficacy of Carboplatin combined with Paclitaxel (Abbreviated chemotherapy) in treating TNBC liver metastases. Compared to control cells, MSLN-deficient HCC1806/HM3 cells showed significantly reduced IC50 values for Paclitaxel and Carboplatin (Fig. 8A), as well as decreased tumor cell viability (Fig. 8B), suggesting that combining MSLN deficiency with Paclitaxel and Carboplatin could be effective in TNBC liver metastases.

Given the limitations of clinical application of MSLN gene knockdown, we employed a pharmacological inhibition strategy using the high-affinity anti-mesothelin monoclonal antibody Amatuximab to target MSLN in orthotopic liver metastasis models established by HCC1806/HM3 cell injection (dosing regimen, Fig. S11A). Notably, Amatuximab-mediated MSLN blockade achieved comparable antitumor efficacy to genetic knockdown (Fig. 8C–E), suggesting a therapeutic potential of pharmacological MSLN inhibition for clinical liver-metastatic TNBC. To comprehensively evaluate therapeutic potential, we further investigated the efficacy of combined chemotherapy with either EGFR inhibition (Erlotinib) or MSLN inhibition (Amatuximab). Compared to the control group, all monotherapies (chemotherapy, Erlotinib or Amatuximab treatment alone) modestly reduced liver metastases and prolonged survival. However, their efficacy differed: the MSLN inhibitor amatuximab demonstrated efficacy comparable to chemotherapy alone, while EGFR inhibition with erlotinib showed slightly weaker efficacy than the other single agents. In contrast, combination therapies significantly enhanced efficacy, with both chemotherapy plus erlotinib and chemotherapy plus amatuximab demonstrating superior effects compared to monotherapies (Fig. 8F–H). Notably, the combination of chemotherapy with amatuximab demonstrated the strongest synergistic therapeutic effect (Fig. 8F–H). Importantly, there was no significant alteration in body weight, relative lung or spleen weight in nude mice (Fig. S11B, C), indicating good tolerability and absence of significant toxicity for these treatments. Taken as a whole, our study demonstrates that the combined use of Carboplatin, Paclitaxel and MSLN inhibition may synergistically inhibit breast cancer liver metastasis, offering therapeutic benefit to TNBC patients with liver metastasis.

## Discussion

Distant metastasis of BC is a primary cause of treatment failure and mortality [27]. Most cases of metastatic breast cancer are associated with liver involvement [28]. Compared to other metastatic sites such as lung and bone, breast cancer liver metastases have poorer survival, with an estimated 5-year overall survival (OS) of only 8.5% [29]. Currently, research on breast cancer metastasis primarily focuses on bone and lung, while the molecular mechanisms of liver metastasis remain incompletely understood [5, 30, 31]. Here, we reveal that MSLN plays is pivotal in liver metastasis of TNBC. MSLN exhibits elevated expression in TNBC hepatotropic metastatic cells, where it interacts with EGFR and activates the downstream ERK1/2 signaling pathway, thereby affecting cell proliferation and viability. Our work addressed that MSLN is a driver of hepatotropic metastatic TNBC. The MSLN/EGFR/ERK1/2 axis represents a potential therapeutic target for TNBC liver metastasis.

Regarding the mechanism of metastasis of malignant tumors, Paget proposed the “seed and soil” theory, where the “seed” refers to tumor cells and the “soil” to the target organ for metastasis [32]. Breast cancer cells, as the “seed”, rely on their genes and proteins, which play crucial roles in distant organ metastasis. For example, GPRC5A has been reported to drive TNBC liver metastasis by activating the mTORC1/p70s6k signaling pathway through recruitment of mTORC1 to the lysosome [33]. Furthermore, our previous study found that RGCC, a lung-specific metastasis driver that promotes TNBC lung metastasis by modulating mitochondrial oxidative phosphorylation [20]. In this work, we identified MSLN as an important regulator and diagnostic factor for TNBC hepatotropic metastasis. Comparative analysis revealed that MSLN levels in TNBC liver metastatic tissues were significantly higher than those in primary TNBC tissues. Additionally, analysis of GEO database data indicated elevated MSLN expression in colorectal cancer liver metastases, but no evidence of its upregulation in other cancer types was found. This observation suggests that the role of MSLN in colorectal cancer liver metastasis warrants further in-depth investigation. Thus, MSLN may serve as a specific biomarker for screening patients with TNBC or colorectal cancer prone to liver metastasis.

The MSLN gene encodes mesothelin, a GPI-anchored protein lacking a kinase domain but capable of activating downstream oncogenic pathways involved in cell proliferation, survival, invasion, and metastasis through unknown mechanisms [34]. It has been reported that GPI-anchored proteins may associate with transmembrane proteins capable of signaling via conventional mechanisms, enabling their downstream effects [35]. Clear links between transmembrane proteins and GPI-anchored proteins have been established. For instance, GPI-anchored glial cell-derived neurotrophic factor receptor-α binds its ligand (GDNF) and subsequently interacts with the transmembrane receptor tyrosine kinase Ret [23]. Similarly, GPI-anchored CD14 (or its complex with LPS) likely binds a transmembrane protein responsible for signal transduction [36]. In this study, we confirmed that MSLN, an elevated protein in liver preferential metastasis-derived cells, interacts with EGFR to activate downstream signaling pathways, thereby promoting cancer metastasis. This highlights the biological and diagnostic significance of the MSLN/EGFR axis in liver metastasis.

EGFR is a transmembrane receptor tyrosine kinase that mediates extracellular signaling to regulate cell survival, proliferation, and differentiation [25]. Previous studies have reported that downstream pathways of EGFR signaling include MAPK-ERK, PI3K-AKT, and JAK-STAT, among others [37]. Activation of EGFR and its downstream pathways is closely related to tumorigenesis and metastasis [38]. For example, EGFR interacts with transmembrane protein 16 A (TMEM16A), activating the JAK/STAT3 and NF-κB pathways, thus promoting tumorigenesis [39]. MUC13 interacts with EGFR to induce PI3K/AKT signaling through EGFR and its internalization inhibition, thus promoting the metastasis of intrahepatic cholangiocarcinoma (ICCA) [40]. Extracellular signal-regulated kinase (ERK) belongs to the mitogen-activated protein kinase (MAPK) family, and the ERK/MAPK signaling pathway is the most well-studied MAPK signaling pathway [41]. Previous studies have demonstrated that MAPK-ERK signaling promotes cell proliferation by controlling the G1/S transition of the cell cycle [42, 43], and regulates apoptotic pathways to promote cell survival [44, 45]. Our study found that the elevated MSLN in hepatic metastatic TNBC could activate the EGFR as well as downstream ERK1/2 signaling pathways, thereby promoting the ability of cancer cells to survive and proliferate in the liver, and further contributing to the formation of hepatic metastases.

Currently, chemotherapy remains the primary first-line treatment for metastatic TNBC; however, chemotherapy resistance poses a significant challenge in managing metastatic cancer [46]. Due to the metastatic heterogeneity of breast cancer, which favors different organs, prognosis and therapeutic responses vary among patients [47]. Our study revealed that the enhanced MSLN in liver-preferential metastatic TNBC cells activates the EGFR-ERK1/2 signaling axis, which enhances cell proliferation and viability, suggesting a potential clinical target for TNBC liver metastases. Importantly, targeting MSLN high expressed liver metastasis breast cancer with a Paclitaxel/Carboplatin combination displayed a significant anti-metastatic effect, suggesting that targeting MSLN-EGFR-ERK signaling could be an effective strategy for treating TNBC liver metastases.

While we have clearly demonstrated the impact of MSLN on the proliferation and survival of metastatic cells, its role in other stages of metastasis, such as intravasation, extravasation, and initial colonization, remains a priority for future investigation by our team. This study primarily employed two animal models: xenografts derived from human TNBC cell lines and murine syngeneic breast cancer transplants. Some other limitations should be acknowledged: (1) intrinsic interspecies divergence in stromal reprogramming between murine models and the human metastatic microenvironment; (2) the inability of immunodeficient hosts (e.g., nude mice lacking functional T cells) to recapitulate the immune-microenvironmental regulation of metastasis observed in humans; (3) potential loss of primary tumor heterogeneity due to prolonged in vitro passaging of cell lines; and (4) abbreviated murine survival windows, which limit the modeling of long-term TNBC liver metastasis progression. Despite these constraints, both models effectively recapitulate critical biological features of human TNBC liver metastasis, including intratumoral intravasation into the circulation, arrest of circulating tumor cells (CTCs) within hepatic sinusoids, endothelial adhesion, basement membrane extravasation, and subsequent formation of micrometastases, culminating in macrometastatic colonization of the liver parenchyma. When translating these findings to clinical contexts, translational caveats merit emphasis: species-specific pharmacokinetic differences, driven by divergent drug metabolism rates, may distort predictions of therapeutic responses. Such disparities could result in agents that appear effective preclinically failing in humans due to accelerated clearance or unanticipated toxicity. To address these limitations, future studies will employ humanized mouse models (reconstituting human immune systems and hepatic microenvironments) in conjunction with organoid-on-chip platforms, aiming to more faithfully simulate human metastatic cascades and enhance preclinical translatability.

## Conclusion

**Fig. 9:**
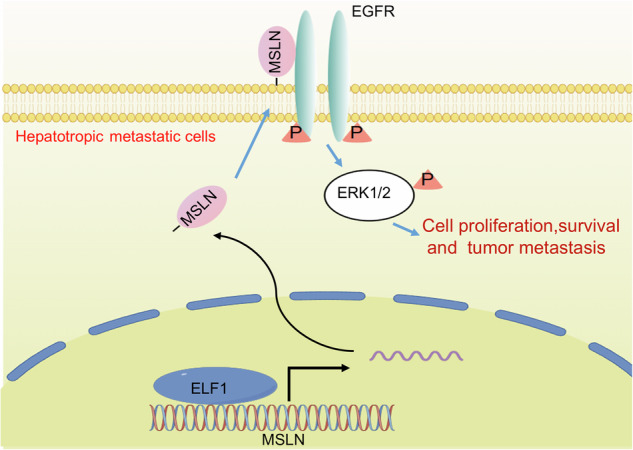
Schematic diagram of MSLN-EGFR-ERK signaling contributing to breast cancer liver metastasis. The transcription factor ELF1 in hepatotropic metastatic cancer cells causes an increased MSLN. The elevated MSLN interacts with EGFR, resulting in phosphorylation of EGFR as well as the activation of downstream ERK1/2 signaling pathway, thereby promoting disseminated TNBC cell survival and proliferation, thus lead to liver metastasis.

MSLN is closely associated with hepatotropic metastasis of TNBC. MSLN may promote liver metastasis by interacting with EGFR and promoting EGFR dimerization, maintaining the EGFR-mediated ERK1/2 signaling pathway to promote TNBC cell proliferation and survival. Therefore, MSLN and its down-stream EGFR-ERK axis could act as a potential therapeutic target for TNBC liver metastasis (Fig. 9).

## Materials and methods

### Clinical tissue specimens

Primary tumor and liver metastasis tissue samples were collected from TNBC patients who provided informed consent at the First Affiliated Hospital of Chongqing Medical University. This study was approved by the Ethics Committee of Chongqing Medical University.

### Cell culture

Human TNBC cell lines (HCC1806, MDA-MB-231, Hs578T, BT-549) and mouse breast cancer cell lines (EMT6, 4T1, E0771, PY81119) and 293 T cell line were purchased from the American Type Culture Collection (ATCC, USA). The cells all passed STR identification and were determined to be free of mycoplasma contamination. PY81119 cells were grown in DMEM/F-12 medium (Gibco, USA) supplemented with 10% fetal bovine serum (FBS) (Gibco, USA) and 1% penicillin/streptomycin (P/S) (MCE, USA). MDA-MB-231, Hs578T, and 293 T were maintained in DMEM (Gibco, USA) with 10% FBS and 1% P/S, while other cell lines were maintained in RPMI 1640 medium (Gibco, USA) supplemented with 10% FBS and 1% P/S. All cell lines were cultured at 37 °C in a 5% CO_2_ incubator.

### To establish the engineered tumor cell models

Lentiviral vectors (NEO-tagged for drug selection) encoding control shRNA and shRNA specifically targeting human *MSLN* and mouse *Msln* were obtained from GenePharma (Shanghai, China). The hepatotropic cells (HCC1806/HM3 and 4T1/HM3) and their control cells (HCC1806/Pri and 4T1/Pri were infected with lentivirus and 5 μg/ml polybrene. Infected cells were selected with 500 μg/ml (Minimum drug concentration capable of total death of non-viral transfected control cells) G418 (Solarbio Life Sciences, China) for two weeks to establish the *MSLM* or *Msln* stably knocked down tumor cells. The target sequences and control shRNAs for human *MSLN* and mouse *Msln* used are provided in Supplementary Table 1. For transient transfection, MSLN-specific siRNA was synthesized by GenePharma (Shanghai, China). Liposome 3000 (invitrogen, LifeTechnologies) was used to transfect cells with siRNA according to the manufacturer’s protocol. The MSLN siRNA is shown in Supplementary Table 1.

Lentiviruses co-expressing human *MSLN* or mouse *Msln* with firefly luciferase (bioluminescence tracking) and G418 resistance (NEO; drug selection) were sourced from GenePharma. Target cells (HCC1806/Pri, 4T1/Pri, BT549, E0771) were transduced to establish stable *MSLN*/*Msln*-overexpressing cells. Infected cells were treated with 500 μg/ml G418 (Solarbio Life Sciences, China) for two weeks to establish target protein stably over-expressed cells.

### Animal experiments

Female BALB/c and C57BL/6j mice (6–8 weeks old) were purchased from the Animal Experiment Center of Chongqing Medical University, while female nude mice (6–8 weeks old) were obtained from GemPharmatech (Chengdu, China). All mice were used in the animal experiments conducted in this study. All animal experiments were conducted with the approval of the Ethics Committee of Chongqing Medical University. Within each strain, mice were randomly assigned to experimental or control groups.

For the organ-specific metastasis mouse model, 100 μl of PBS containing 1 × 10⁶ HCC1806 (nude mice) or 4T1 (BALB/c mice) cells was injected in situ into mammary fat pad of mice. Cells from primary tumors and organ metastases were separately isolated and cultured (cells isolated from mouse primary tumors were named HCC1806-Pri or 4T1-Pri, the metastatic tumor cells isolated from liver, brain and lung organs were named HM, BM and LM, respectively). After stable clones were established, organ metastatic cells were reinoculated into new mice to generate metastatic tumors. After at least three rounds of screening, highly organotropic metastasis cells were successfully established, designated as HCC1806/HM3 or 4T1/HM3 (liver-preferential metastases), HCC1806/BM3 or 4T1/BM3 (brain-preferential metastases), and HCC1806/LM3 or 4T1/LM3 (lung-preferential metastases). The firefly luciferase reporter gene (for bioluminescence tracking) retrovirus was infected with 4T1/HCC1806-derived cells and puromycin (1 μg/mL, Sigma Aldrich, USA) was selected for 14 days.

For the experimental metastasis mice models, 50 μl of PBS suspension containing 1 × 10⁶ cells (luc + ) was injected into the spleens of mice: HCC1806-derived cells and BT549 into nude mice, 4T1-derived cells into BALB/c mice, and E0771 into C57BL/6j mice. When mice were observed to be in a weak state, all mice were given an intraperitoneally injected of D- luciferin substrate (150 mg/kg), after 10 min, the mice were euthanized, livers were imaged using bioluminescence imaging (BLI), and liver metastasis was analyzed. Parallel groups of mice were monitored for 2–3 months, with survival recorded and analyzed.

For MSLN knockdown efficacy studies, HCC1806/HM3 cells with stable MSLN knockdown or control cells (1 × 10⁶ cells in 100 μL PBS) were orthotopically injected into the mammary fat pads of nude mice. Liver metastasis served as the primary endpoint. For pharmacological inhibition, HCC1806/HM3 cells (1 × 10⁶ cells in 100 μL PBS) were similarly injected. When xenografts reached ~50 mm³, tumor-bearing mice received intravenous injections of 60 mg/kg Amatuximab or control IgG (MCE, USA) every three days. Liver metastasis was assessed as the primary endpoint. When mice were observed to be in a weak state, in vitro liver bioimaging and survival analysis were performed as described above, lung, and spleen tissues were harvested, weighed.

For combination therapy evaluation, HCC1806/HM3 cells (1 × 10⁶ cells in 100 μL PBS) were orthotopically injected into nude mice. When the primary tumor xenograft was observed to reach approximately 50 mm^3^, mice were treated intraperitoneally every three days with:10 mg/kg Paclitaxel (MCE, USA) and 5 mg/kg Carboplatin (MCE, USA), 60 mg/kg Amatuximab (MCE, USA), 50 mg/kg Erlotinib (EGFR tyrosine kinase inhibitor; MCE, USA), administered alone or in combination according to experimental groups. Metastasis and survival were evaluated as described above.

Data collection and analysis were performed by investigators blinded to the experimental groups. Group allocations were concealed by labeling all animals and samples with ear tag numbers only, with the treatment key held by an independent colleague until final analysis.

### Western blotting analysis(WB)

Cells were lysed in RIPA buffer (Solarbio Life Sciences, China) supplemented with PMSF (MCE, USA) to extract total protein, which was quantified using a BCA protein assay kit (Beyotime, China). Denatured protein samples were separated by SDS-PAGE and transferred onto PVDF membranes (Bio-Rad, USA). After blocking with 5% skim milk at room temperature, membranes were incubated overnight at 4 °C with specific primary antibodies, washed with PBS, and incubated with HRP-conjugated secondary antibodies (Biosharp, China) for 90 minutes at room temperature. Proteins were detected using BeyoECL Plus (Beyotime, China). The antibodies are listed in Supplementary Table 2. Grayscale values of target proteins were quantified using ImageJ with loading normalization achieved by dividing target protein values by corresponding internal reference protein densities (e.g., GAPDH or β-actin).

### EGFR dimerization assay

Primary tumor cell control and MSLN-overexpressing cells were treated with or without 50 ng/mL EGF (Beyotime, China) at 37 °C for 30 min. Cells were washed with ice-cold DPBS and maintained on ice. Subsequently, cells were incubated with 3 mM BS³ crosslinker (bis(sulfosuccinimidyl) suberate) (Thermo Fisher, China) in ice-cold DPBS for 20 min. After washing with cold DPBS, proteins were extracted and subjected to Western blot analysis using anti-EGFR antibody.

### Flow apoptosis assay

Annexin V-APC/7-AAD Apoptosis Detection Kit (KG1106; KeyGEN BioTECH) was used. Briefly, cells were harvested with EDTA-free trypsin, washed with PBS and resuspended, incubated with 5 μL of Annexin V-APC and 5 μL of 7-AAD staining buffer for 10 min at room temperature in the dark, and the cell death index was determined by flow cytometry. Annexin V-FITC/PI Apoptosis Detection Kit (KG1102; KeyGEN BioTECH) was used as described above.

### RNA preparation, qRT-PCR, and RNA sequencing

Total RNA was extracted using TRIZOL reagent (Invitrogen) and reverse transcribed with the PrimeScript RT kit (Takara, Japan). Quantitative real-time PCR was conducted using the SYBR Premix Ex Taq II kit (Takara, Japan) and the CFX96 real-time PCR detector. Primer sequences are provided in Supplementary Table 3. RNA sequencing was conducted by BGI (Shenzhen, China).

### Dual-luciferase reporter assay and chromatin immunoprecipitation (ChIP) assay

The pGL3-MSLN wild-type (WT) and mutant (MUT) reporter gene vectors, along with the pcDNA3.1 vector expressing ELF1, and siRNA targeting ELF1 were produced by GenePharm (Shanghai, China). Cells were seeded on 96-well plates were co-transfected using transfection reagent: (1) For 293 T and HCC1806-P cells: pcDNA3.1( + )-ELF1 (or control vector), pGL3-MSLN WT/MUT reporter, and pRL-TK Renilla vector; (2) For HM3 cells: ELF1-targeting siRNA (or control siNC), pGL3-MSLN WT, and pRL-TK; (3For 4T1-P cells: pcDNA3.1( + )-ELF1 (or control vector), pGL3-MSLN WT, and pRL-TK). The activities of firefly and Renilla luciferase were evaluated 48 h post- transfection, using a dual luciferase reporter gene detection kit (Beyotime,) according to the manufacturer’s instructions.

The Pierce Sepharose ChIP kit (Thermo Fisher Scientific, USA) was utilized with ELF1 and control IgG antibodies. The enriched DNA samples were analyzed by qPCR. ChIP primer sequences can be found in Supplementary Table 4.

### HE and Immunohistochemistry (IHC) staining

Paraffin-embedded livers were sectioned, dewaxed and rehydrated. Sections were stained using an H&E staining kit (Solarbio Life Sciences, China). For IHC, a universal two-step test kit (ZSGB-BIO, China) was used, and the experiment was performed according to the manufacturer’s instructions as follows: After dewaxing and hydration, tissue slides were washed with PBS and subjected to antigen retrieval. The slides were then incubated 60 min at 37 °C with the primary antibody, followed by a 20-min incubation with the secondary antibody. Visualization was performed using DAB, and the slides were counterstained with hematoxylin. IHC staining was scored following previous methods [48].

### Immunoprecipitation and LC–MS/MS

Cells were lyzed on ice using Western and IP lysates supplemented with PMSF (P0013, Beyotime), followed by centrifugation at 12,000 revolutions per minute for 30 min. The supernatant was collected and immunoprecipitated with the specified antibodies, then incubated overnight at 4 °C. The immunoprecipitated complexes were combined with magnetic protein A/G beads (HY-K0202, MCE) and incubated for 2 h. After washing, the immunoprecipitated proteins were subjected to western blotting analysis.

For LC-MS/MS analysis, a reaction solution (2.5% SDS/100 mM Tris-HCl, pH 8.5) was added to the immunoprecipitated magnetic bead samples and incubated 10 min at 95 °C. After centrifugation, the supernatant was collected, and proteins were precipitated using the TCA method. The protein pellet was dissolved in a reconstitution solution and incubated 30 min at 60 °C to complete reduction and alkylation. The sample was diluted with an equal volume of ddH_2_O, trypsin was added at an enzyme-to-protein mass ratio of 1:50, and the mixture was incubated at 37 °C overnight with shaking for enzymatic digestion. After desalting, the digested peptides were injected through an autosampler and analyzed using an UltiMate 3000 RSLCnano tandem Q Exactive HF mass spectrometer (Thermo). Raw mass spectrometry data were analyzed using MaxQuant (2.2.0.0), with the Andromeda algorithm built into the software. The search was conducted against the Human Protein Sequence Database (20240807) from UniProt. Search results were filtered with a 1% false discovery rate (FDR) threshold applied at both protein and peptide levels.

### Multiplex immunofluorescence (MxIF)

For MxIF staining, cell slides were processed using a double-labeled three-color multiplex fluorescence staining kit (Aifang Biotechnology, China). Cells were fixed on glass slides, blocked with BSA, and incubated overnight with a primary antibody at 4 °C. After incubation with the HRP-Polymer immunohistochemical secondary antibody at room temperature for 30 min, fluorescent dye was added for 3–10 min, and the antibody was eluted. Subsequent staining rounds involved blocking, primary and secondary antibody incubation, and fluorescent dye addition. Sections were subsequently stained with DAPI (Aifang Bio) and sealed with an anti-fluorescence quencher. Antibodies used include rabbit anti-human/mouse MSLN, rabbit anti-human/mouse EGFR (Supplementary Table 3), and HRP-Polymer goat anti-rabbit immunohistochemical secondary antibody (Aifang Bio). Fluorescent dyes used include TYR-570 and TYR-690 (Aifang Bio). Fluorescence signals were captured and recorded using KFBIO KF-FL-020. Manders’ colocalization coefficients (MCC) for MSLN and EGFR were quantified using the Colocalization Finder plugin in ImageJ.

### Transwell assay

A total of 2 × 10⁴ 4T1-derived cells, and 1 × 10^5^ HCC1806-derived cells were suspended in 200 μL of serum-free medium. For invasion assays, cells were plated in 8 μm Transwell inserts (Labselect, China) pre-coated with Matrigel (Corning, USA). For migration assays, cells were placed in uncoated upper chambers. The lower chambers were filled with 600 μL of medium supplemented with 10% FBS. The Transwell membranes were fixed and stained after 24 h of incubation. and the cells that migrated or invaded were quantified.

### Cell proliferation, cell viability assay and colony formation assay

Cell proliferation was assessed, and cell viability was measured using the Cell Counting Kit-8 (CCK8, Yensen, China) following the manufacturer’s protocol. For proliferation and viability assays, cells were seeded in 96-well plates at each number of densities and cultured for the indicated time. Cell growth and survival were measured using the CCK8 following the manufacturer’s protocol. For colony formation assays, cells were seeded into six-well plates at densities of 1000 cells/well and incubated at 37 °C for a week, while HCC1806-derived cells (2000 cells/well) were cultured for 14 days. All genetically modified cell lines (MSLN knockdown/overexpression) and their corresponding controls were maintained for identical durations. Then colonies were fixed with methanol for 15 min and stained with crystal violet for 20 min.

### Molecular docking

The protein structures of human MSLN and EGFR were collected in the PDB database, and the structures of EGFR-P00533 and MSLN-Q13421 were docked by HEX8.0 software (https://hex.loria.fr/). Finally, the best docking group was selected and analyzed by pymol mapping.

### Data collection

MSLN and ELF1 expression-related BC survival curves obtained from the Kaplan–Meier plotter online site. Microarray data used in this study were obtained from GEO database with accession numbers GSE58812, GSE72718, GSE76180, GSE67675 and GSE4611. In situ gene expression and clinical correlation data of human breast cancer were obtained from TCGA. Organ metastasis data were obtained from MET500 Cohort.

### Statistical analysis

In cases where the data conformed to a normal distribution, paired t-tests and unpaired Student’s t tests were applied for two-group comparisons, while one-way ANOVA was used for comparisons among multiple groups. Overall survival curves were calculated using the Kaplan–Meier method and compared using the log-rank test. Correlations were assessed using the Pearson correlation coefficient, and cell proliferation curves were analyzed using two-way ANOVA. All analyses were conducted using GraphPad Prism 9.0. The data were expressed as the mean ± standard deviation (SD. Lack of statistical significance was denoted as ns, while statistical significance was defined as *P* < 0.05. *P* values are shown as follows: *, *P* < 0.05; **, *P* < 0.01; ***, *P* < 0.001.

## Supplementary information

Supplementary Information file

Western Blots file

## Data Availability

The RNA-seq data have been deposited in the Gene Expression Omnibus (GSE306125 and GSE306126).
