# Supplementary material for: MSLN-mediated activation of EGFR-ERK1/2 signaling drives liver metastasis in breast cancer
Source: Cell Death Discov. 2026 Jan 9;12:11. doi: 10.1038/s41420-025-02835-9 (PMC12789440; doi:10.1038/s41420-025-02835-9)
Supplement: Supplementary file 1 — Supplementary Information file [file 41420_2025_2835_MOESM1_ESM.docx]

**Supplementary Information file**

**
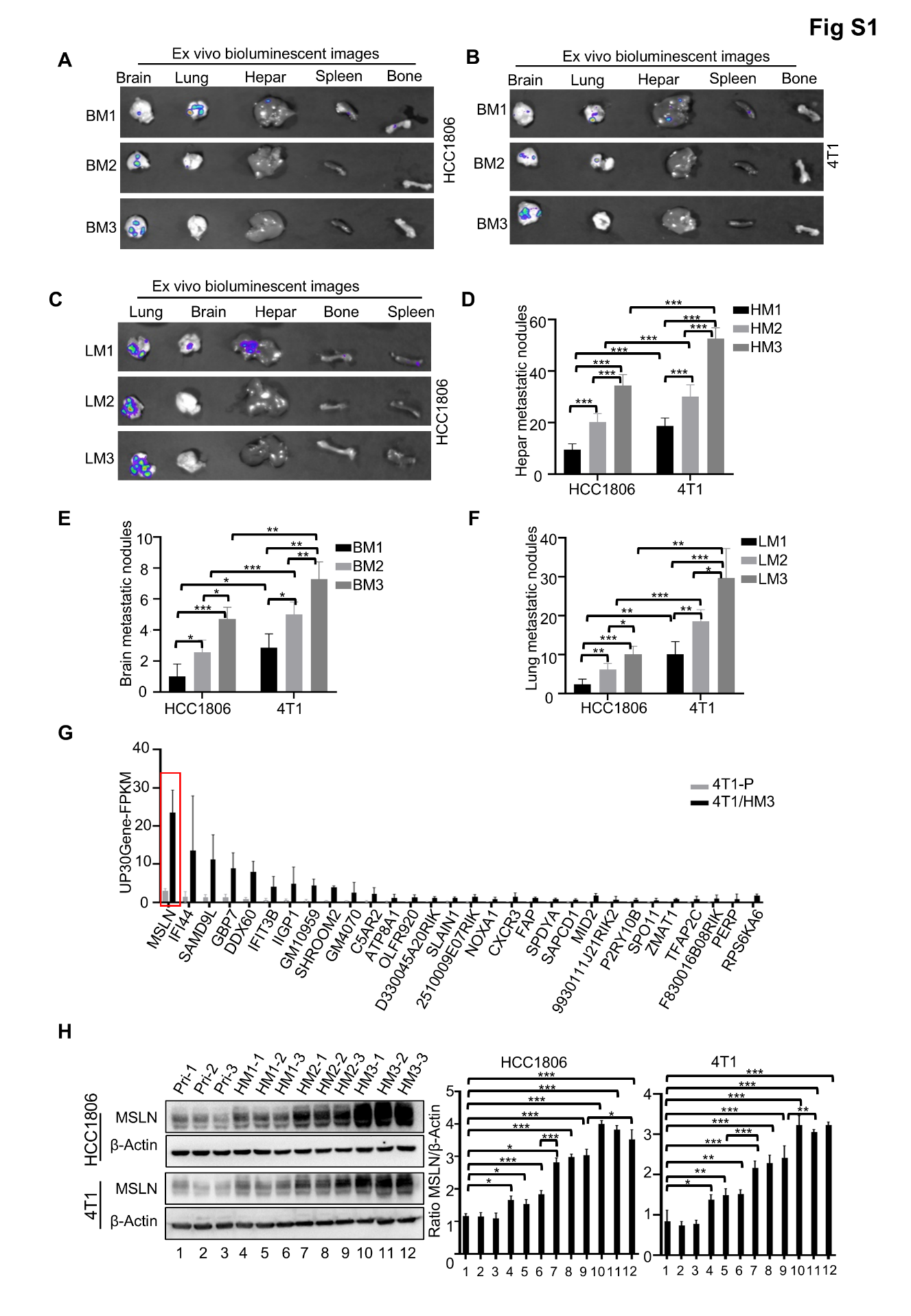
Supplementary figures**

**Fig S1 Related to result 1**

**A-B.** Representative ex vivo bioluminescence images of HCC1806 (A) and 4T1 (B) cell brain-tropic metastasis in major organs. **C.** Representative ex vivo bioluminescence images of lung-tropic metastasis of HCC1806 cells in major organs. **D-F.** Statistics on the number of metastatic nodules in major organs (hepar, brain, lung) in organ metastasis model (n=7). **G.** Histogram show the FPKM of the top 30 upregulated genes for RNA sequencing in 4T1/HM3 and 4T1/Pri cells. **H.** MSLN protein levels in the specified cells were determined by WB(n=3). (The data are presented as the mean ± SD). (*P < 0.05, **P < 0.01, ***P < 0.001).

**
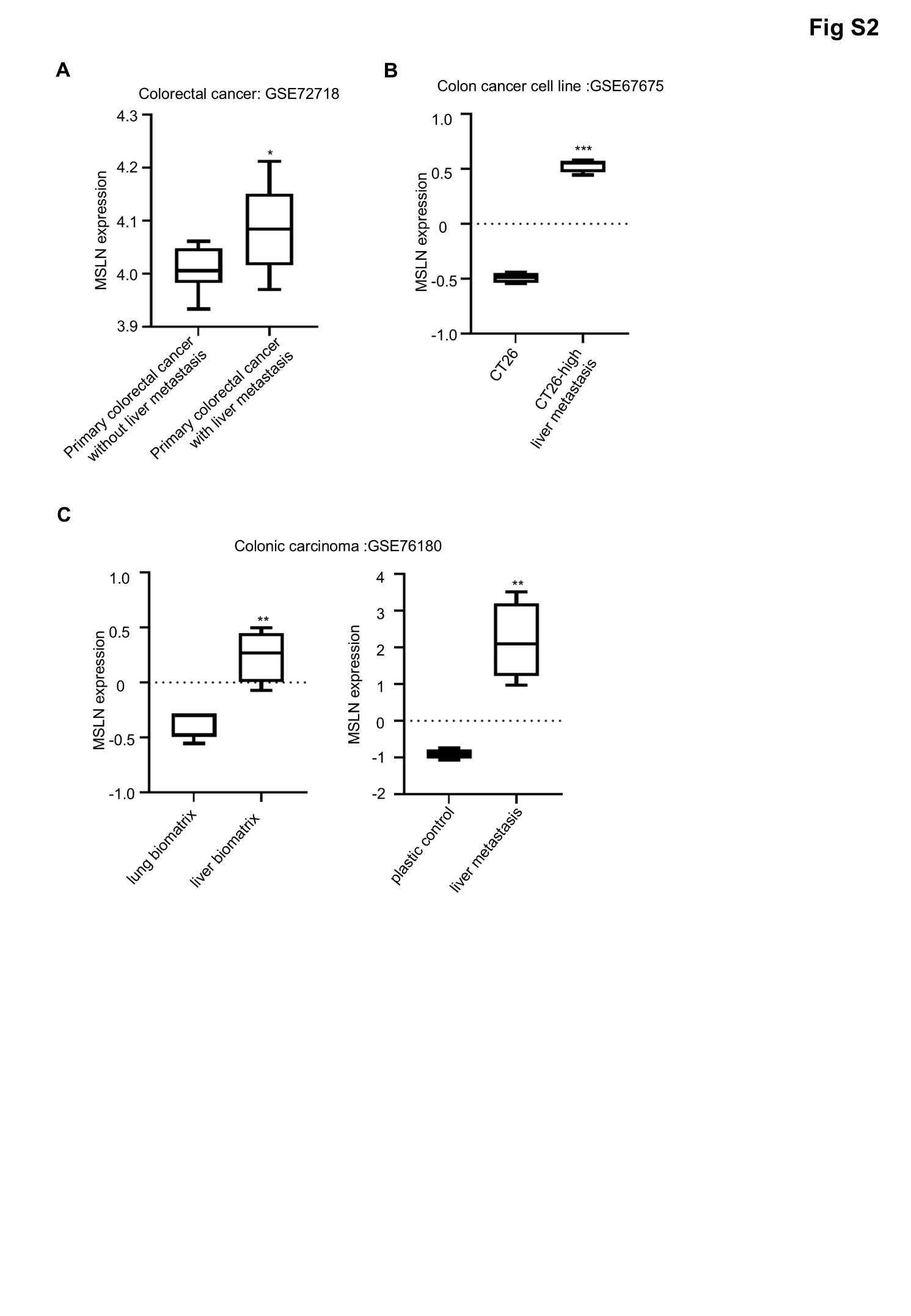
**

**Fig S2 Related to result 1**

**A-C.** Elevated MSLN mRNA expression was in liver metastases compared to other sites, derived from microarray datasets of metastatic colorectal cancer from different data sources. (P < 0.05, **P < 0.01, ***P < 0.001).

**
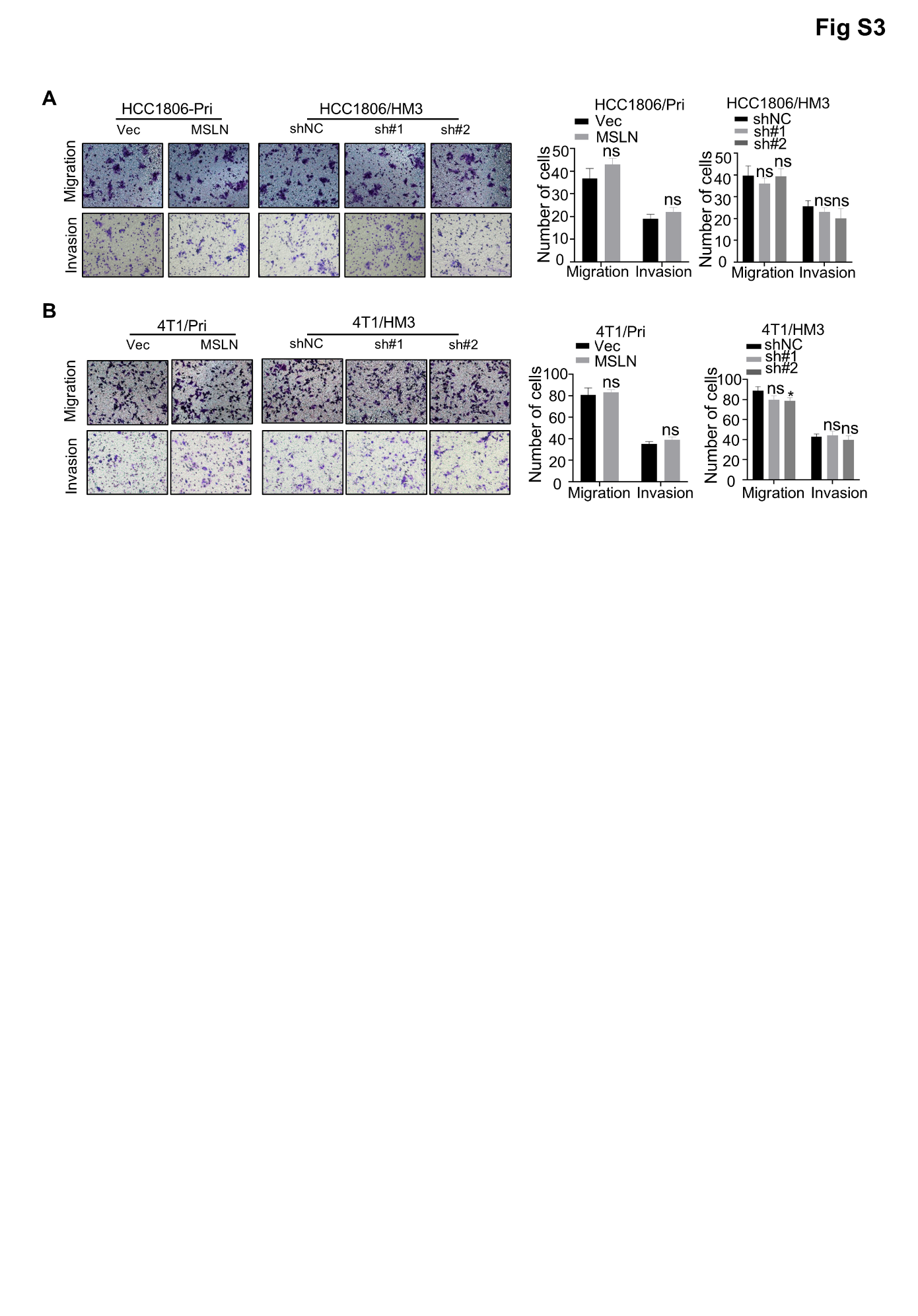
**

**Fig S3 Related to result 2**

**A-B**. Migration and invasion abilities of ectopic MSLN overexpressing and control Pri cells and knockout MSLN or control treated HM3 were determined using Transwell assays. (The data are presented as the mean ± SD; ns: no statistical difference, *P < 0.05).


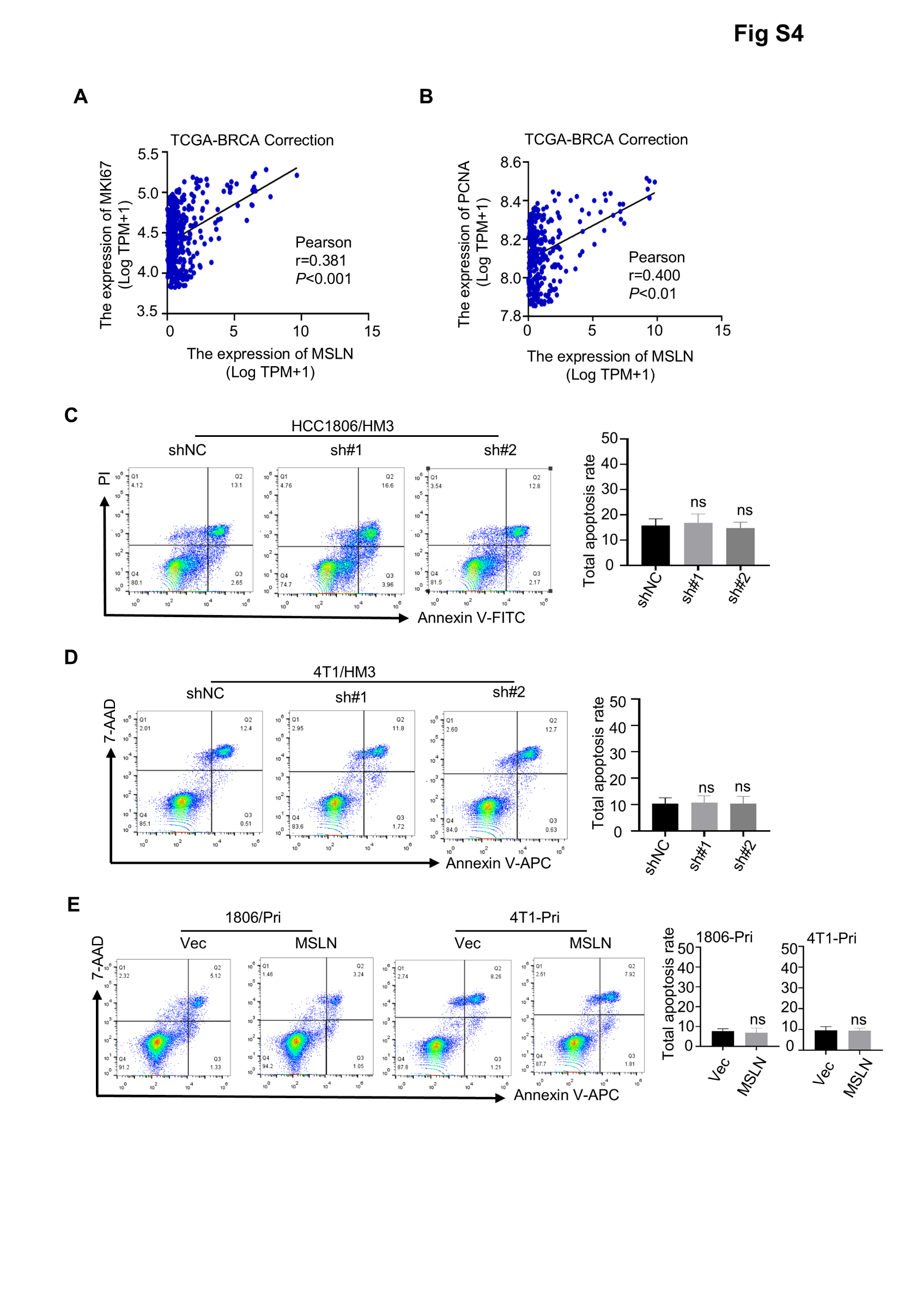


**Fig S4 Related to result 2**

**A-B.** Clinical data from TCGA-BRCA showed that MSLN was positively correlated with proliferative activity-related Marker Ki67 (A) and PCNA (B) expression. **C-E**. The effect of MSLN on apoptosis was detected by flow cytometry and the total apoptosis rate was counted. (The data are presented as the mean ± SD, ns: no statistical difference,).


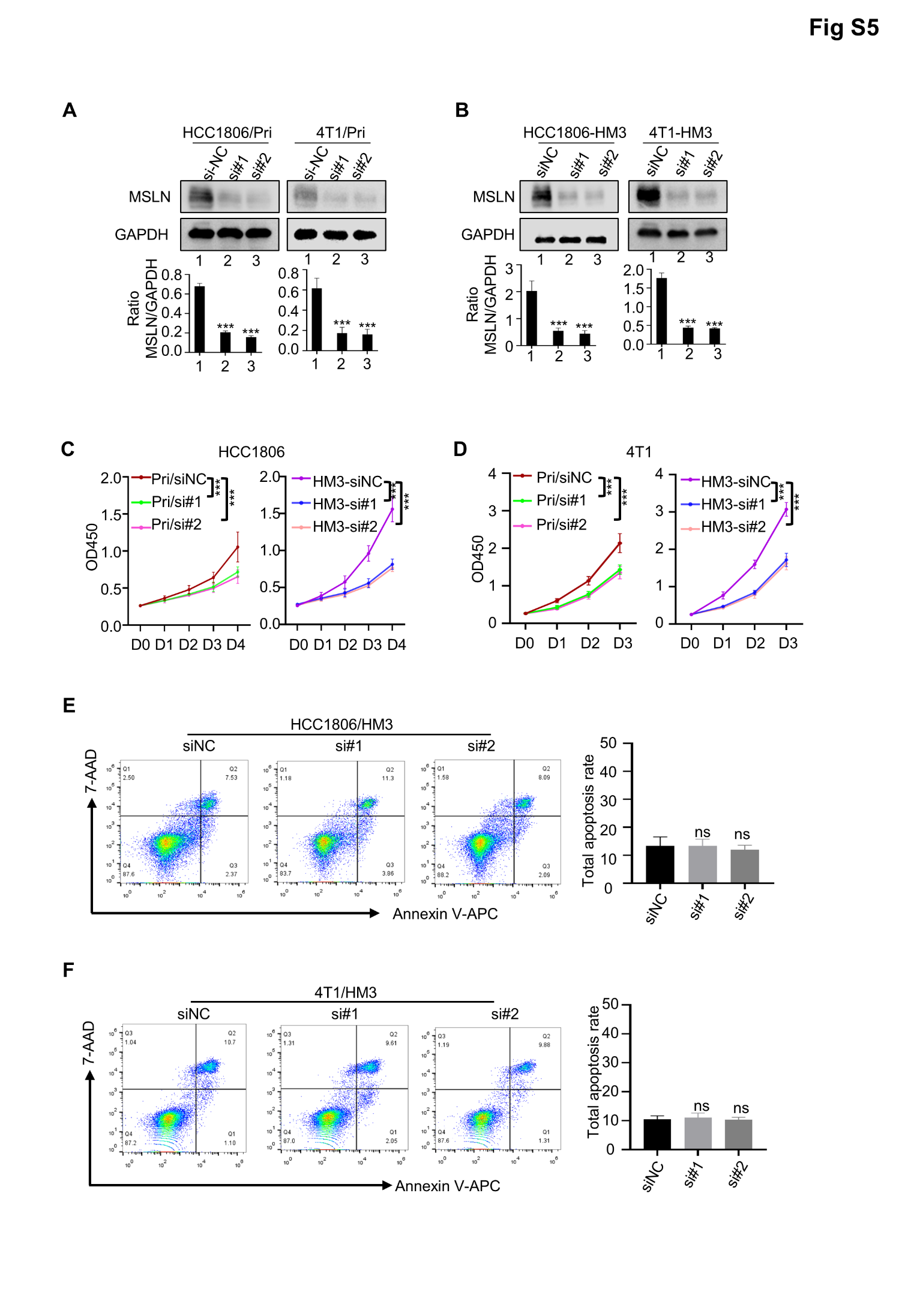


**Fig S5 Related to result 2**

**A-B.** Transiently knocked down MSLN in HCC1806/HM3, 4T1/HM3 and corresponding Pri cells, and the knockdown efficiency was detected by WB validation (n=3). **C-D.** CCK-8 assay was used to evaluate the proliferation of cells (n=6). **E-F.** Flow cytometry was used to evaluate the effect of MSLN on apoptosis of HM3 cells (n=3). (*#*, *MSLN*) (The data are presented as the mean ± SD; ns: no statistical difference, ***P < 0.001).


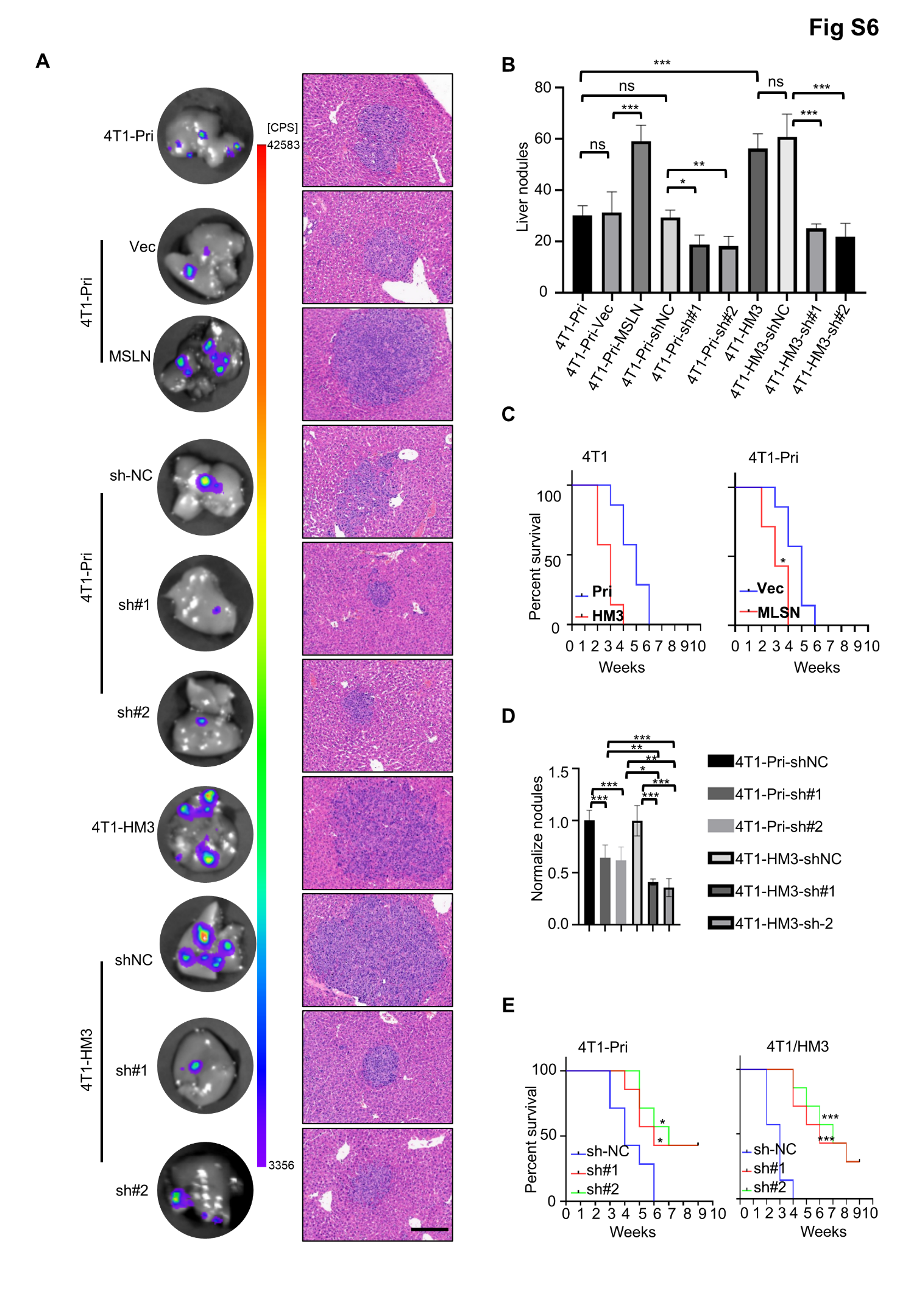


**Fig S6 Related to result 2**

**A-B.** Mouse spleen injections with luc-indicator cells (4T1-derived cells), when mice were observed to be in a weak state, mice were given an intraperitoneally injected of D- luciferin substrate (150 mg/kg), yielded representative in vitro bioluminescence imaging (BLI) of liver and H&E images of liver metastases (A) (scale bar, 200 μm), liver metastatic nodule count statistics (B). **C.** Kaplan-Meier Survival Curves of Indicated Cell-Injected Mice (n = 7). **D.** The number of liver metastasis nodules in MSLN-knockdown groups was normalized to their respective control groups to compare the inhibitory efficacy of MSLN knockdown on metastasis in Pri and HM3 cell lines. **E.** Kaplan-Meier Survival Curves of Indicated Cell-Injected Mice (n=7). (*#*, *MSLN*) (Data are presented as the mean ± SD; ns: no statistical difference, **P < 0.01, ***P < 0.001).


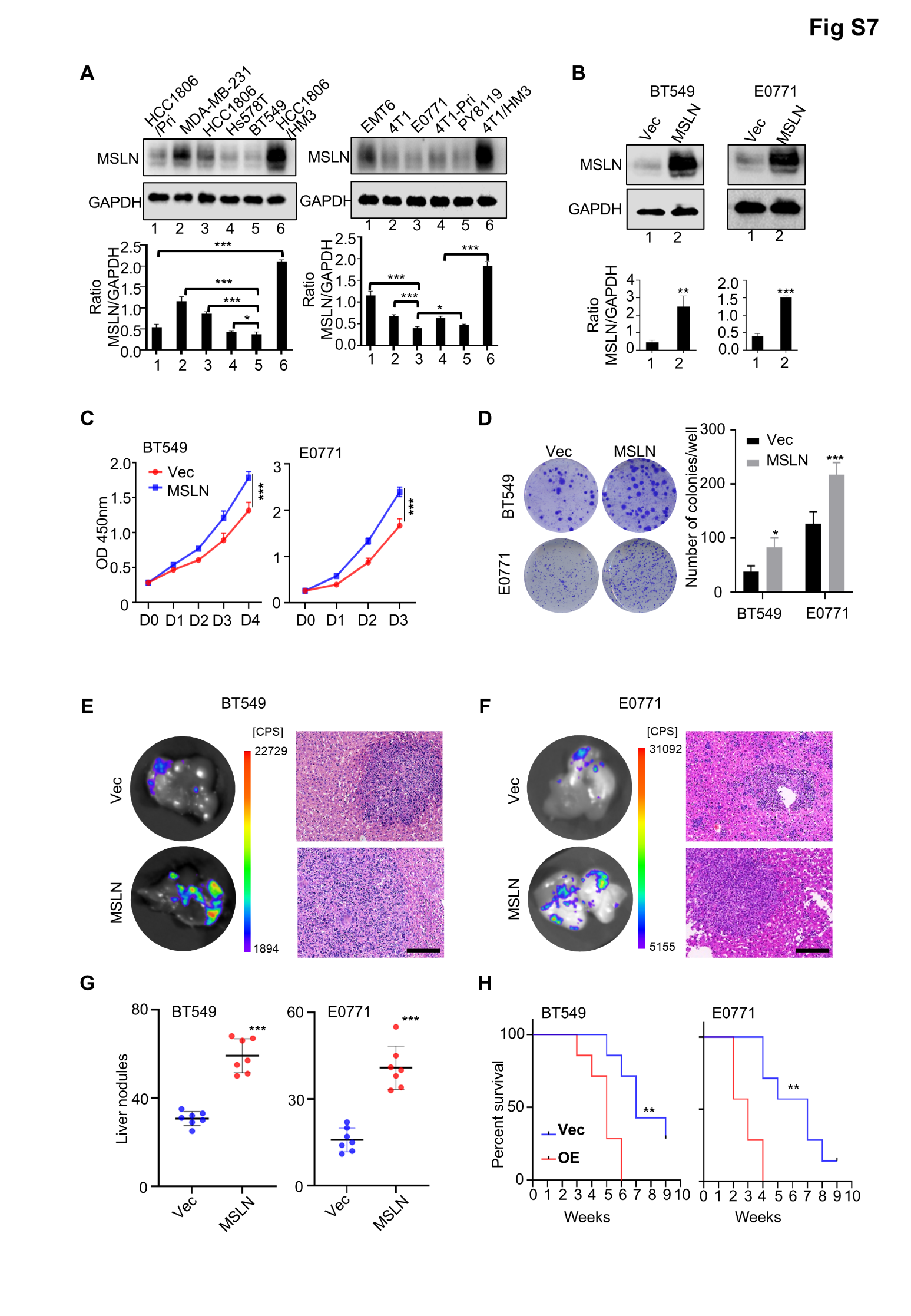


**Fig S7 Related to result 2**

**A.** WB detects MSLN expression in HCC1806-derived cells and several wild-type TNBC cells used in the study (left), and in 4T1-derived cells and several wild-type mouse breast cancer cells (right) (n=3). **B.** Efficiency of WB detection of overexpressed MSLN in BT549 cells and E0771 cells (n=3). **C-D.** CCK-8 (C) and colony formation assays (D) were used to evaluate the proliferation and survival ability of TNBC cells. **E-H.** Mouse spleen injections with luc-indicator cells, when mice were observed to be in a weak state,, mice were given an intraperitoneally injected of D-luciferin substrate (150 mg/kg), yielded representative in vitro bioluminescence imaging (BLI) of liver and H&E images of liver metastases (E-F) (scale bar, 200 μm), liver metastatic nodule count statistics (G) and Kaplan-Meier Survival Curves of Indicated Cell-Injected Mice (n=7) (H). (Data are presented as the mean ± SD; *P < 0.05, **P < 0.01, ***P < 0.001).


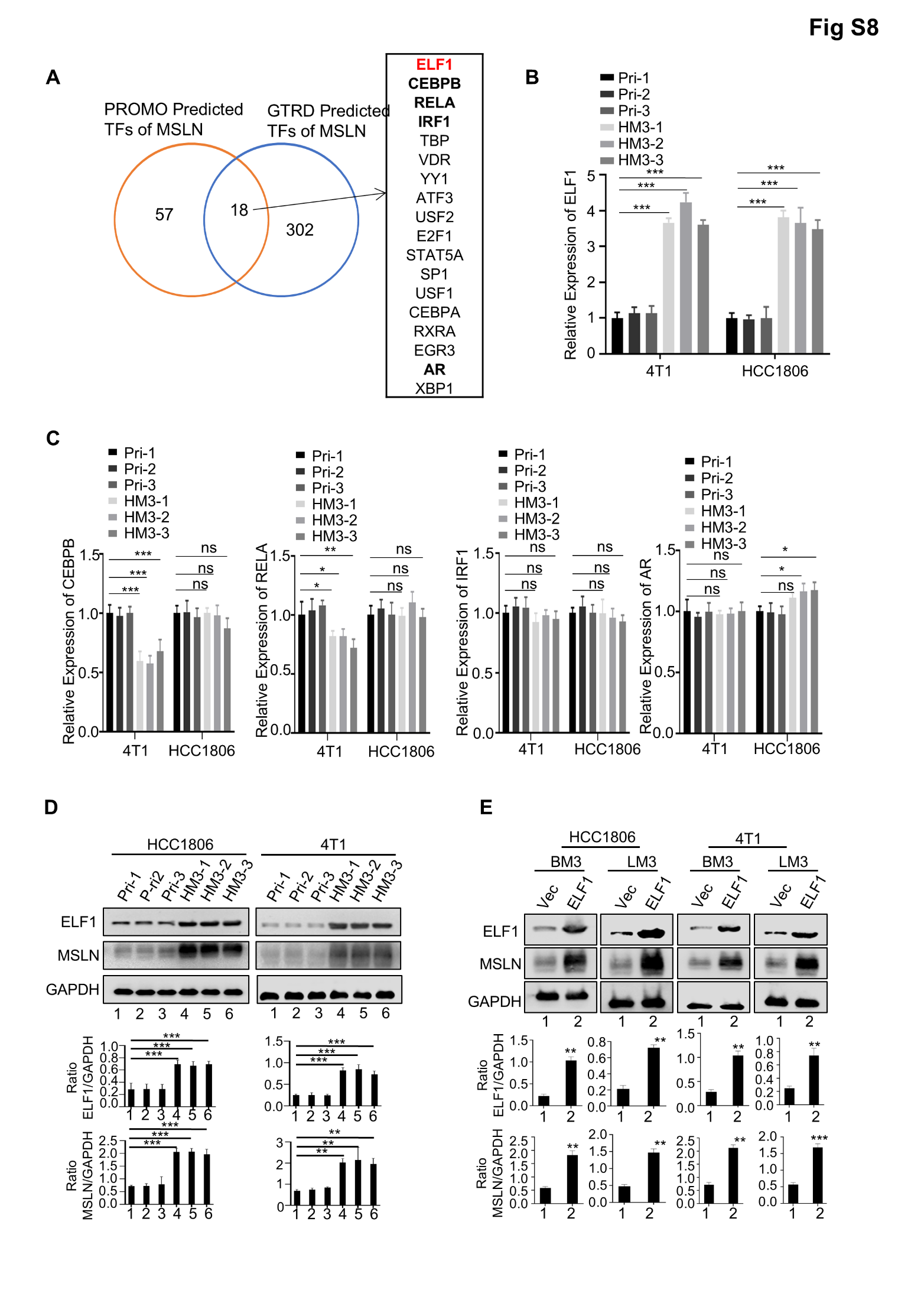


**Fig S8 Related to result 3**

**A.** Venn diagram of overlapping transcription factors (TFs) between TFs predicted by the PROMO database and the GTRD database that potentially regulate MSLN expression. **B-C.** The mRNA levels of transcription factors (ELF1, CEBPB, RELA, IRF1 and AR) associated with MSLN expression in Pri and HM3 cells were analyzed using qRT-PCR. **D.** ELF1 protein levels in Pri and HM3 cells were determined by WB (n=3). **E.** The regulatory ability of ELF1 on MSLN gene expression in BM3 and LM3 metastatic cells was detected by WB (n=3). (Data are presented as the mean ± SD; ns: no statistical difference, *P < 0.05, **P < 0.01, ***P < 0.001).


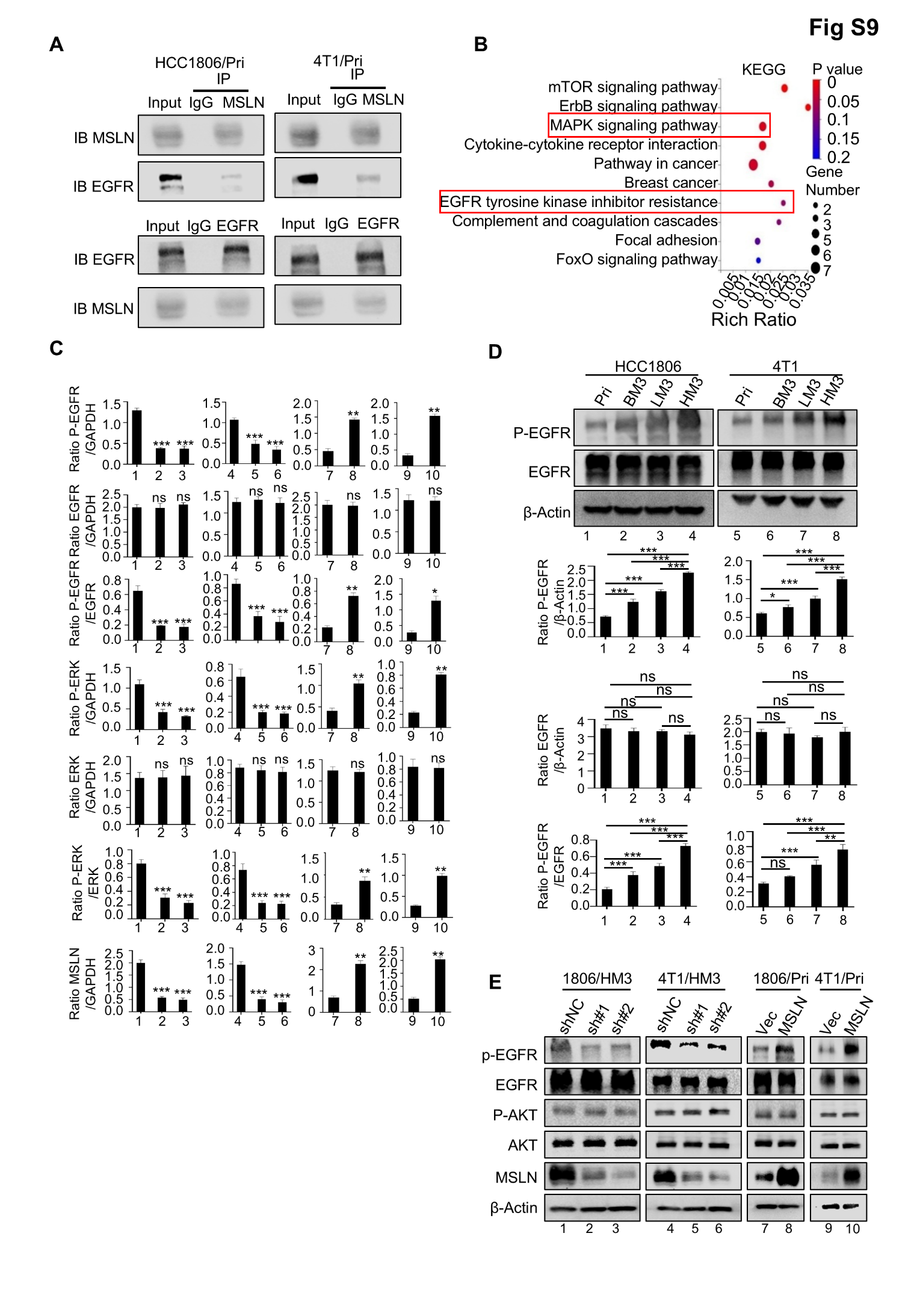


**Fig S9 Related to result 4**

**A.** The direct interaction between MSLN and EGFR in HCC1806/Pri (B) and 4T1/Pri cells was verified by Co-IP assays using anti-MSLN and anti-EGFR antibodies. **B.** KEGG pathways enriched by the DEGs between MSLN-knockdown HCC1806/HM3 cells and control cells. **C.** Normalized gray value statistics were performed on the WB results in Fig. 6G (n=3). **D.** WB analysis was performed on the phosphorylation levels of cells with different organo-tropic metastases of 4T1 and HCC1806 and corresponding Pri cells (n=3). **E.** Western blot analysis of the key factors expressions of the EGFR-PI3K/AKT signaling in cells with MSLN knockdown (HCC1806/HM3 and 4T1/HM3, left panels) and overexpression (HCC1806/Pri and 4T1/Pri, right panels) (*#*, *MSLN*). (Data are presented as the mean ± SD; *P < 0.05, **P < 0.01, ***P < 0.001).


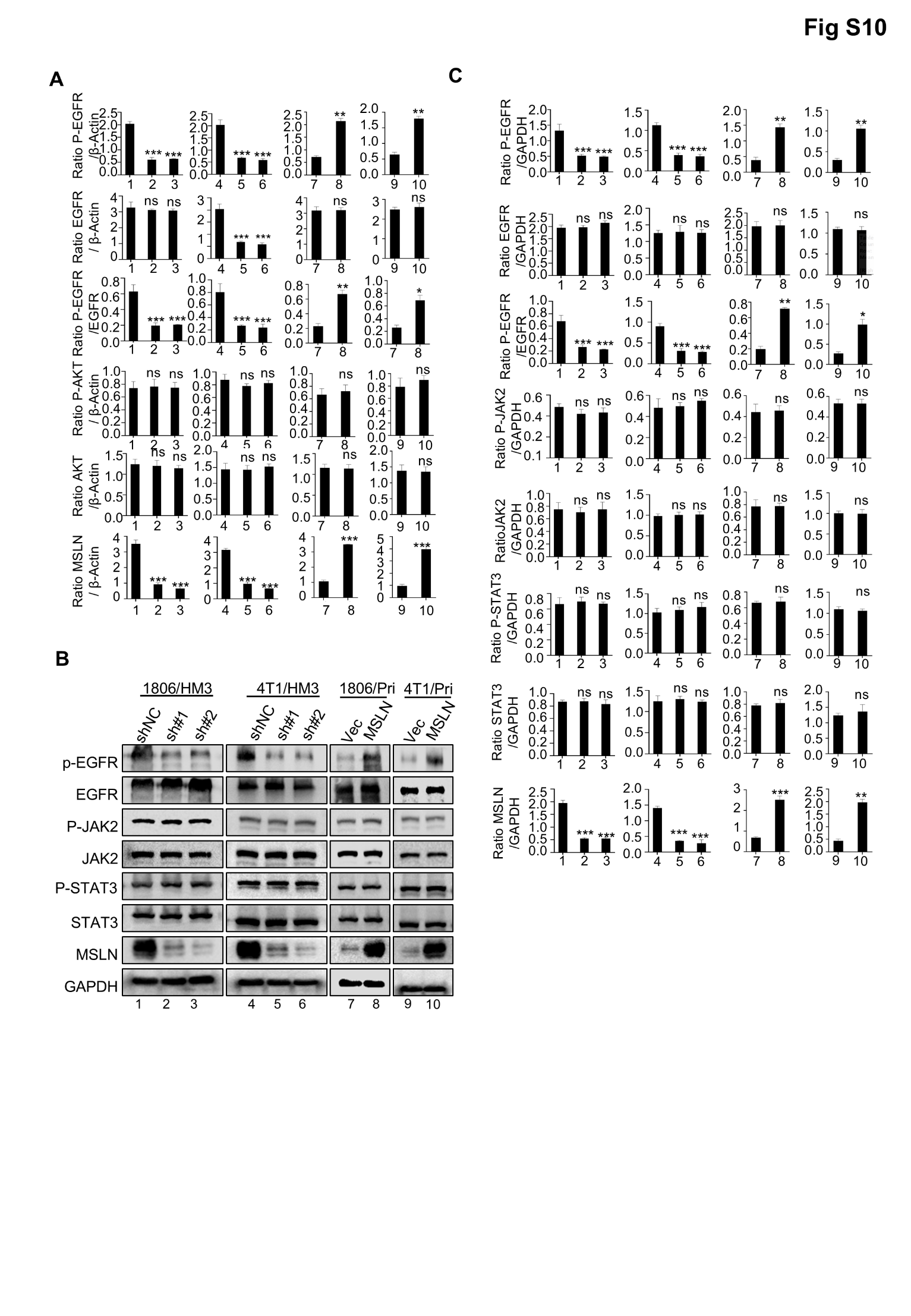


**Fig S10 Related to result 4**

**A.** Normalized gray value statistics were performed on the WB results in Fig. S9E (n=3). **B-C**. Western blot analysis of the key factors expressions of the EGFR-JAK/STAT3 signaling in cells with MSLN knockdown cells and overexpressing cells, and standardized gray value statistics were performed (n=3) (*#*, *MSLN*). (Data are presented as the mean ± SD; *P < 0.05, **P < 0.01, ***P < 0.001).


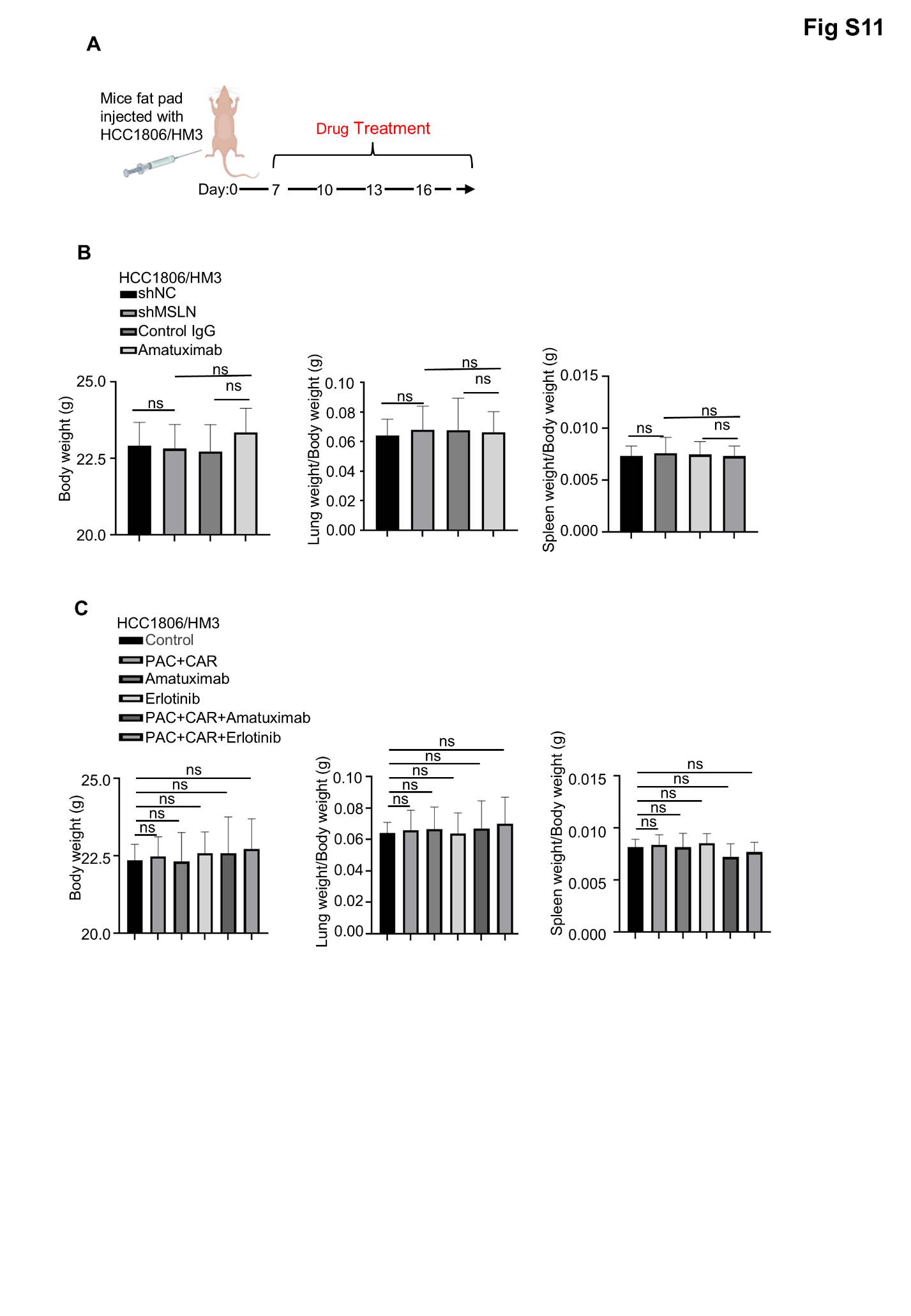


**Fig S11 Related to result 6 A.** Experimental therapeutic strategy for mice. **B-C.** Graphs depicting body weight, lung-to-body weight ratios, and spleen-to-body weight ratios in mice (n=7 mice/group). (Data were presented as mean± SD; ns: no statistical difference).

**Supplementary Tables**

**Supplementary Table 1. Sequences of shRNA and siRNA**

| **Target gene** |  | **Sequences** |
| --- | --- | --- |
| MSLN shNC |  | TTCTCCGAACGTGTCACGT |
| MSLN (human) shRNA1 |  | TGGCCAACCCACCTAACATTT |
| MSLN (human) shRNA2 |  | CTGGACGTCCTAAAGCATAAA |
| MSLN (mouse) shRNA1 |  | TGTGACCTGCCTGGGAAATTT |
| MSLN (mouse) shRNA2 |  | CTGGGTCACTTCTTCAGATAT |
| siNC |  | SS: UUCUCCGAACGUGUCACGUTT  AS: ACGUGACACGUUCGGAGAATT |
| MSLN (human)siRNA1 |  | SS: ACUUCUUGUAGAAGAUGAGGC  AS: CUCAUCUUCUACAAGAAGUGG |
| MSLN (human)siRNA2 |  | SS: GCUAGACAAAGACACCCUAGA  AS: UAGGGUGUCUUUGUCUAGCUG |
| MSLN (mouse)siRNA1 |  | SS: GACCCAGACUACAAAGACAAG  AS: UGUCUUUGUAGUCUGGGUCUG |
| MSLN (mouse)siRNA2 |  | SS: CAAGGAUGUCAAAGCUGAAUG  AS: UUCAGCUUUGACAUCCUUGGG |
| ELF1 (human)siRNA1 |  | SS: AGCAAAUUCAAAUACUAGGUC  AS: CCUAGUAUUUGAAUUUGCUAG |
| ELF1 (human)siRNA2 |  | SS: GGAUGUUGCUGAAGAAGAAAU  AS: UUCUUCUUCAGCAACAUCCAG |
| ELF1 (mouse)siRNA1 |  | SS: AACUAUUGAGAAUAUCAGCAC  AS: GCUGAUAUUCUCAAUAGUUAU |
| ELF1 (mouse)siRNA2 |  | SS: CGAUCAAGAGUAUCUUCAAGU  AS: UUGAAGAUACUCUUGAUCGGU |

**Abbreviation**: SS, Sense Strand. AS, Antisense Strand.

**Supplementary Table 2. Antibodies used in the current study**

| **Primary antibody** | **Product** | **Catalog#** |
| --- | --- | --- |
| Rabbit anti-MSLN | Abcam | ab187063 |
| Rabbit anti-MSLN | HUABIO | HA721810 |
| Rabbit anti-MSLN | Abcam | ab196235 |
| Rabbit anti-Mesothelin | Abmart | PC25346 |
| Rabbit anti-EGFR | Abmart | T55112 |
| Rabbit anti-P-EGFR | Abmart | T55232 |
| Rabbit anti-ELF1 | Abmart | PA4896 |
| Rabbit anti-ERK1/2 | Abmart | T40071 |
| Rabbit anti-p-ERK1/2 | Abmart | TA1015 |
| Rabbit anti-AKT | Abmart | T55561 |
| Rabbit anti-p-Akt | Abmart | TP56018 |
| Rabbit anti-JAK2 | HUABIO | ET1607-35 |
| Rabbit anti-p-JAK2 | HUABIO | ET1607-34 |
| Rabbit anti-STAT3 | HUABIO | ET1607-38 |
| Rabbit anti-p-STAT3 | HUABIO | ET1603-40 |
| Mouse anti-GAPDH | proteintech | 60004-1-IG |
| Rabbit anti-IgG | proteintech | 30000-0-AP |

**Supplementary Table 3.** **Primer sequences for qRT-PCR**

| **Gene** | **Primer** | **Sequence** |
| --- | --- | --- |
| H-MSLN | Forward (5’ to 3’) | TTGGACCTGCTGCTATTCCT |
|  | Reverse (5’ to 3’) | GTGATGCGGGAGAAGAAACG |
| M-MSLN | Forward (5’ to 3’) | GACCTGAAGACCGAGGAGGA |
|  | Reverse (5’ to 3’) | CCCTGAAGTCCCAAACCCAG |
| H-β-actin | Forward (5’ to 3’) | TGGCACCCAGCACAATGAA |
|  | Reverse (5’ to 3’) | CTAAGTCATAGTCCGCCTAGAAGCA |
| M-β-actin | Forward (5’ to 3’) | CATCCGTAAAGACCTCTATGCCAAC |
|  | Reverse (5’ to 3’) | ATGGAGCCACCGATCCACA |
| H-ELF1 | Forward (5’ to 3’) | GGATGAACGACAGCTTGGTG |
|  | Reverse (5’ to 3’) | ATTGGGCTCTTCCACACAGG |
| M- ELF1 | Forward (5’ to 3’) | ATCAAGTCGGAATCAAGC |
|  | Reverse (5’ to 3’) | TGGGTAGGATAGGGAGC |
| H-CEBPB | Forward (5’ to 3’) | TGACGCAGCGGTTGCTA |
|  | Reverse (5’ to 3’) | CGGCTCTGACTCGCTAAAGT |
| M-CEBPB | Forward (5’ to 3’) | TTGATGCAATCCGGATCAAACG |
|  | Reverse (5’ to 3’) | CAGTTACACGTGTGTTGCGTC |
| H-RELA | Forward (5’ to 3’) | AACCTGGGAATCCAGTGTGTG |
|  | Reverse (5’ to 3’) | CCCACGCTGCTCTTCTTGGAA |
| M-RELA | Forward (5’ to 3’) | GGCAGTGACGCGACGA |
|  | Reverse (5’ to 3’) | TGAGGGGAAACAGATCGTCCA |
| H-IRF1 | Forward (5’ to 3’) | CAAATCCCGGGGCTCATCTG |
|  | Reverse (5’ to 3’) | TGCTTTGTATCGGCCTGTGT |

**Continued supplementary Table 3.** **Primer sequences for qRT-PCR**

| **Gene** | **Primer** | **Sequence** |
| --- | --- | --- |
| M-IRF1 | Forward (5’ to 3’) | TGAACAGTCTGAGTGGCAGC |
|  | Reverse (5’ to 3’) | CCCATCAGGAGGTTTCCTCG |
| H-AR | Forward (5’ to 3’) | CTTCGCCCCTGATCTGGTTT |
|  | Reverse (5’ to 3’) | CTCATTCGGACACACTGGCT |
| M-AR | Forward (5’ to 3’) | ATCTGCTGCGTATTGTGGCT |
|  | Reverse (5’ to 3’) | TTTGCTTGGTTGGCACACAG |

**Supplementary Table 4. Primer sequences used for PCR analysis in ChIP assay**

| **Gene name** | **Primer sequences** |
| --- | --- |
| MSLN | F: 5’-GCAGATTCCGTACTGGGGAT-3’  R: 5’-GAGCTGTCTTCCCTACGCAT-3’ |

**Supplementary Table 5. Eleven potential interaction sites between MSLN and EGFR proteins**

| **EGFR** | **MSLN** | **Interaction:** |
| --- | --- | --- |
| ARG677 | GLN43 | Hydrogen bonding |
| GLU27 | ARG64 |  |
| GLU685 | GLU734 |  |
| GLU685 | GLN505 |  |
| ARG776 | GLN100 |  |
| ARG776 | GLU99 |  |
| ILE688 | GLN100 |  |
| GLU317 | ARG413 |  |
| GLU317 | ARG414 |  |
| ARG832 | ARG161 |  |
| SER695 | ALA184 |  |
