# Supplementary figures and images for: MSLN-mediated activation of EGFR-ERK1/2 signaling drives liver metastasis in breast cancer
Source: Cell Death Discov. 2026 Jan 9;12:11. doi: 10.1038/s41420-025-02835-9 (PMC12789440; doi:10.1038/s41420-025-02835-9)

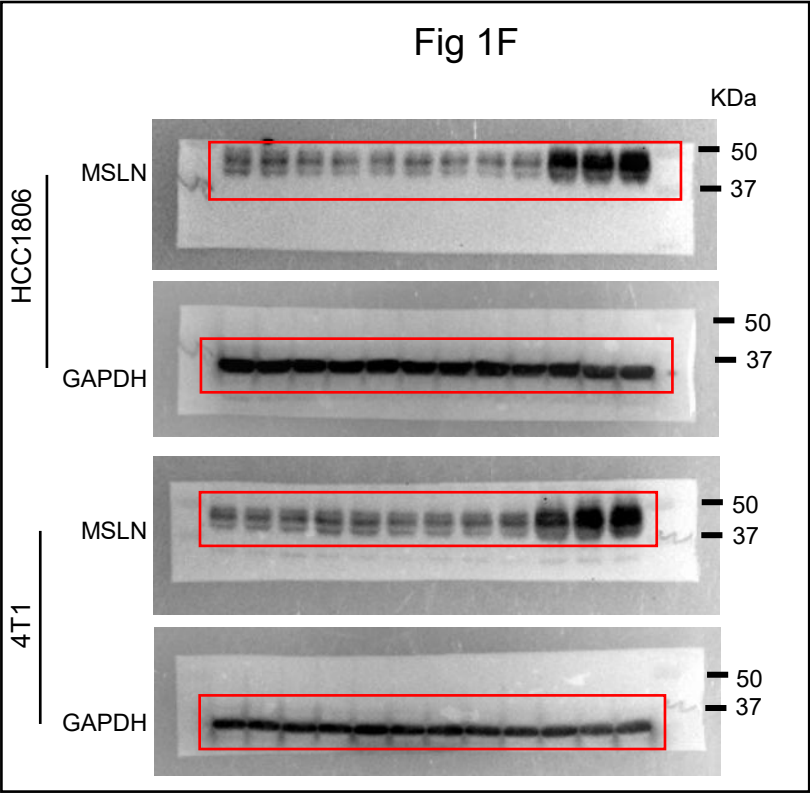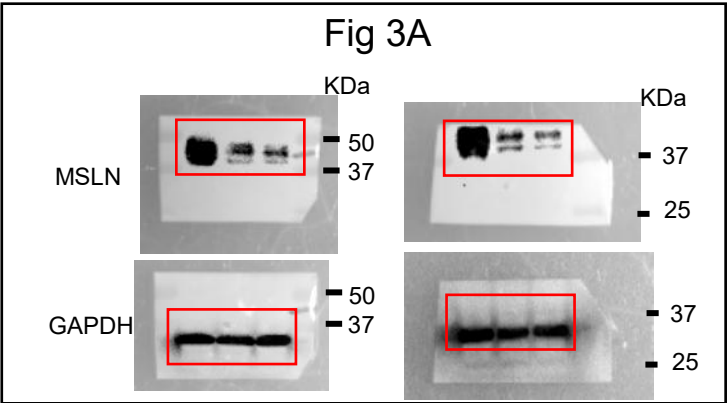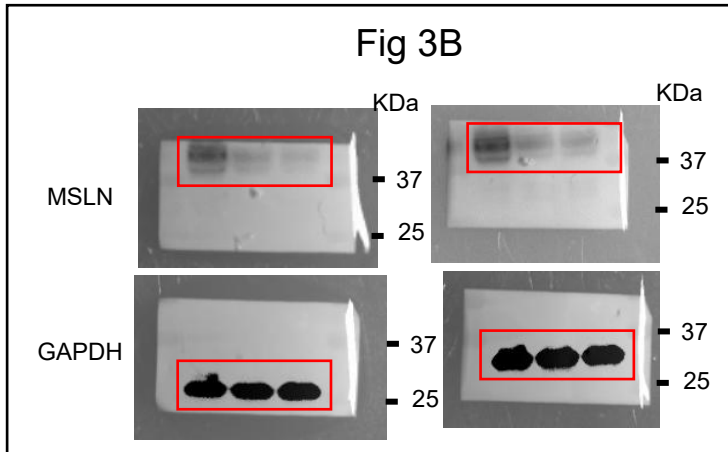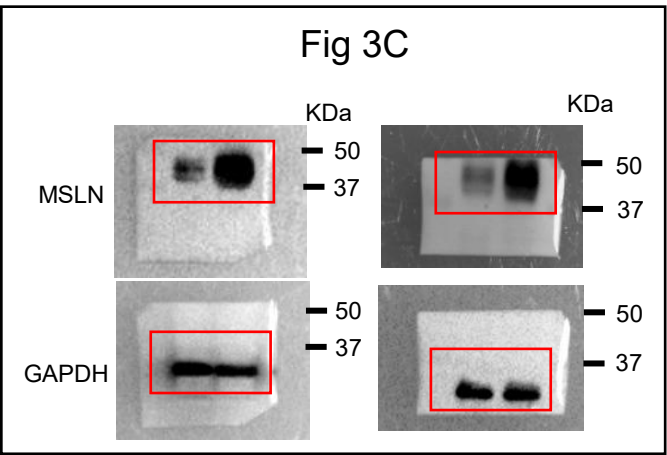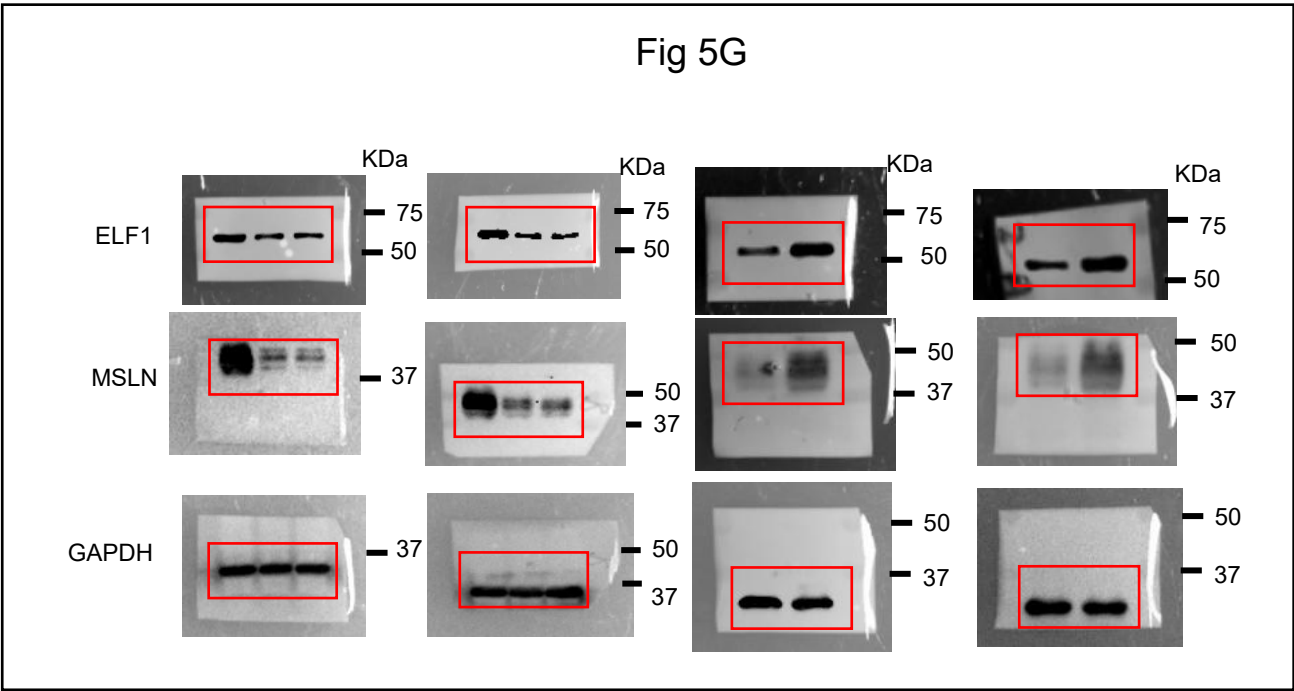

Fig 6B

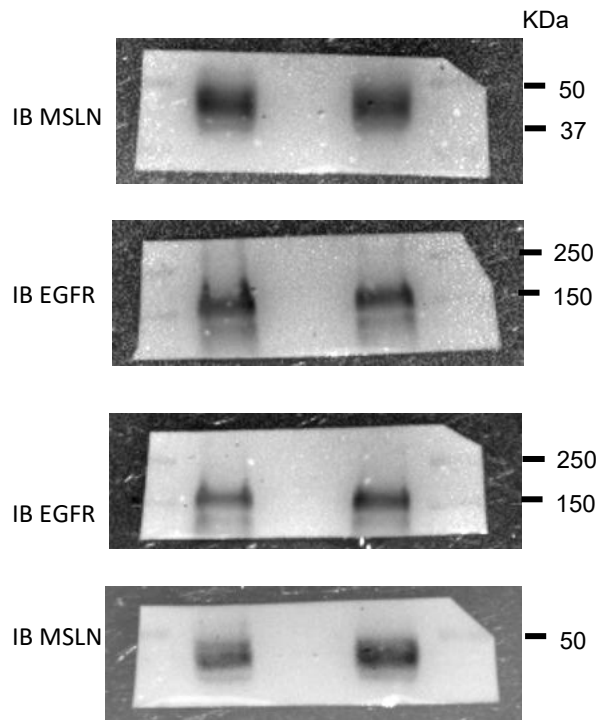

Fig 6C

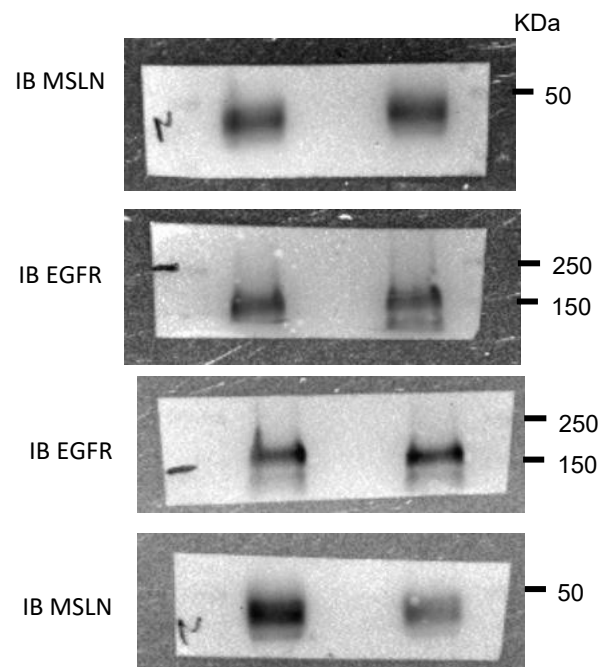

Fig 6G

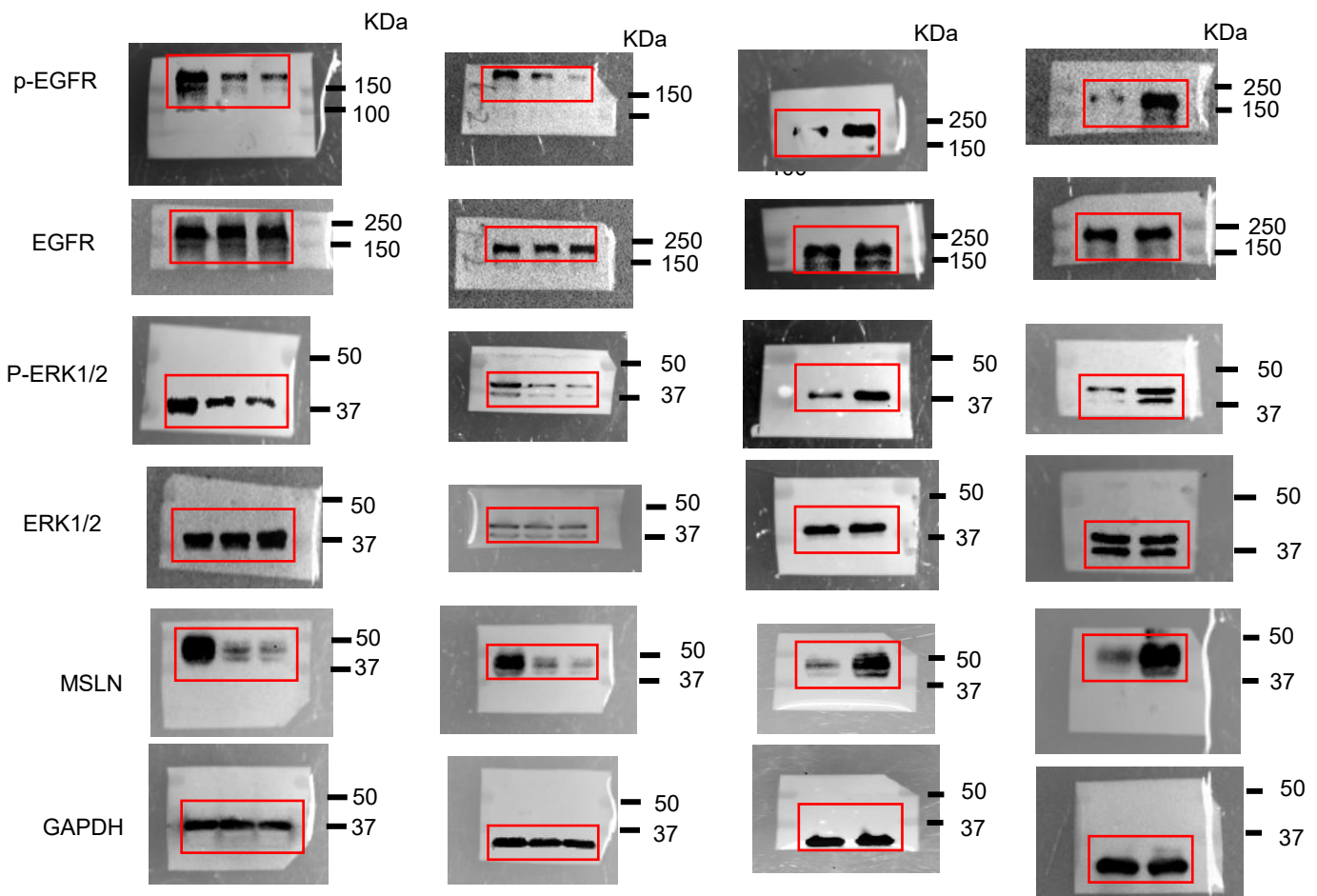

Fig 6H

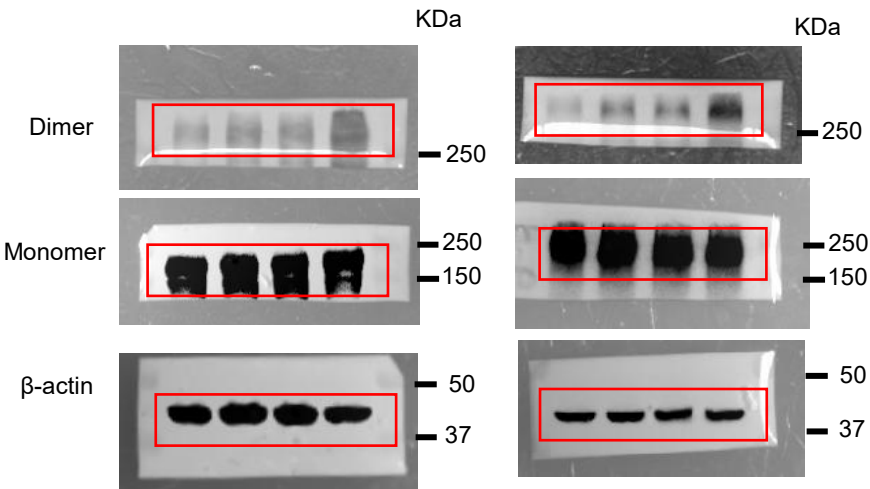

Fig 7A

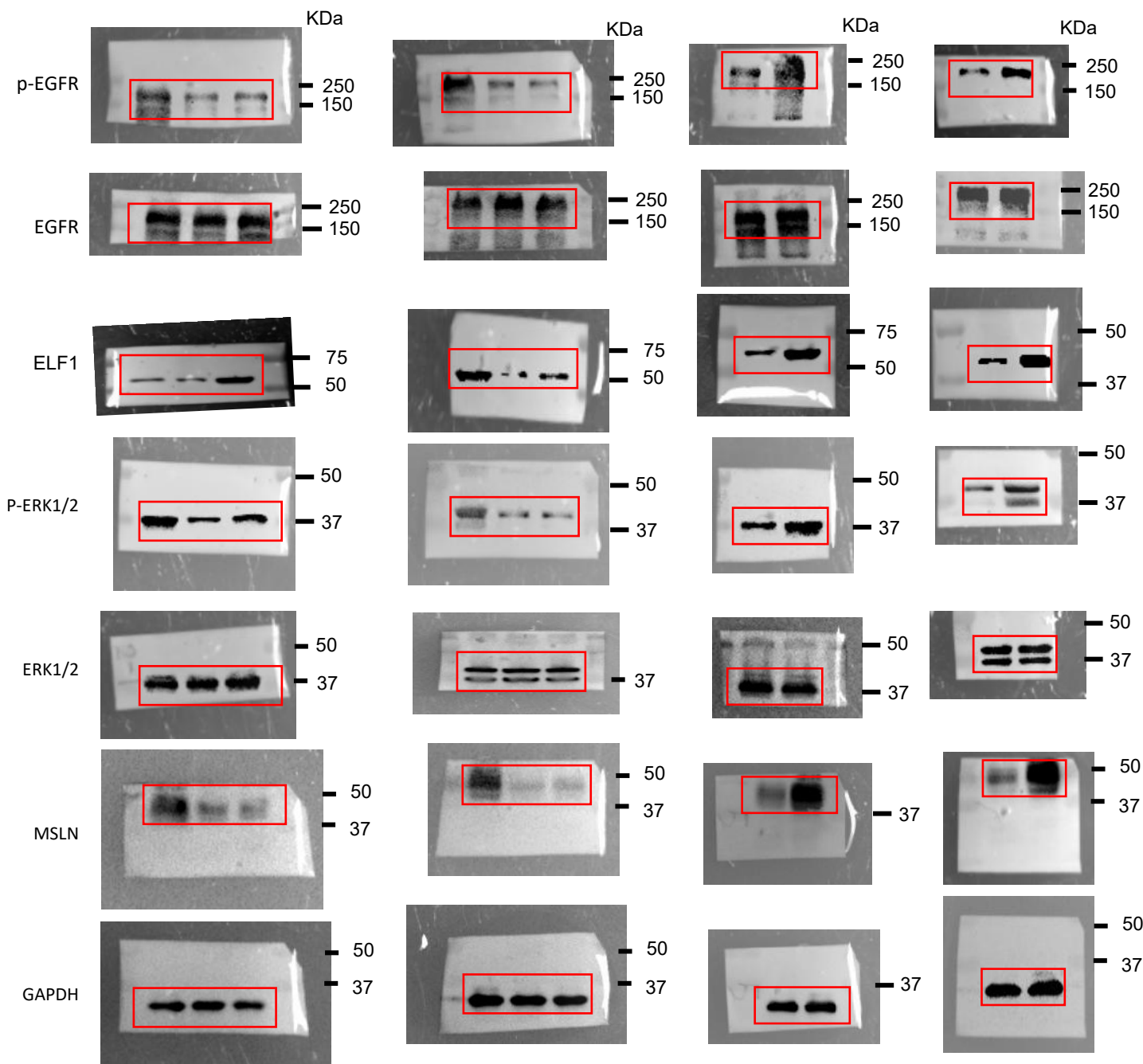

Fig S1H

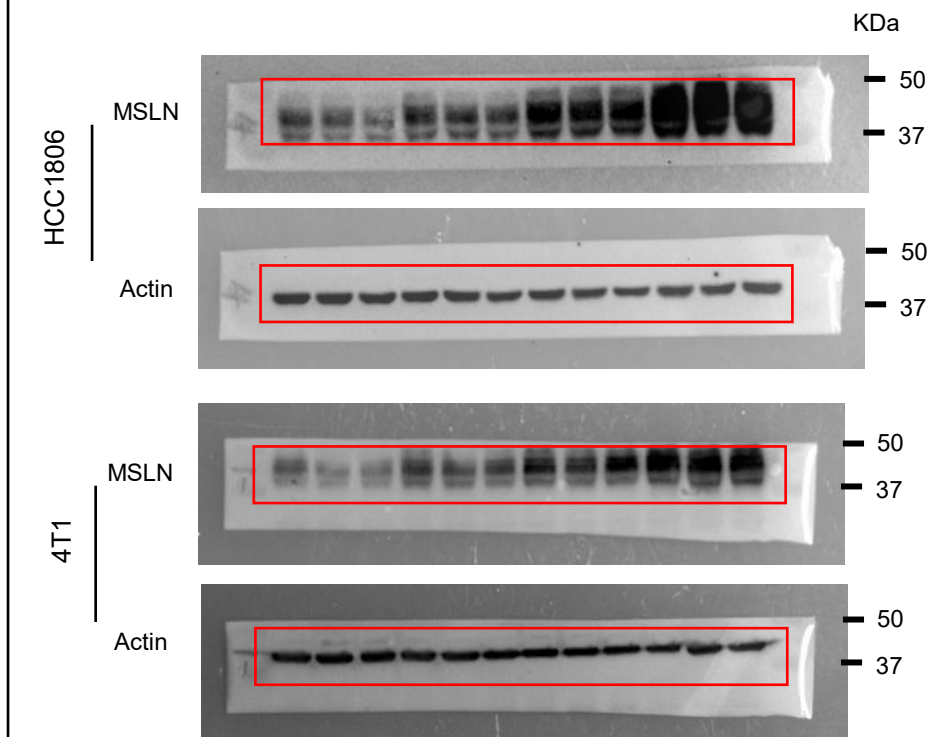

Fig S5A

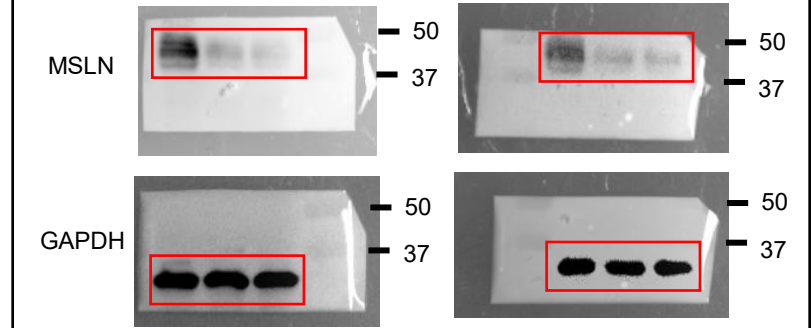

Fig S5B

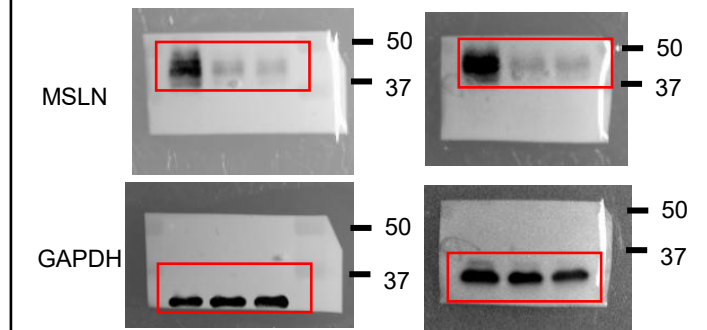

Fig S7A

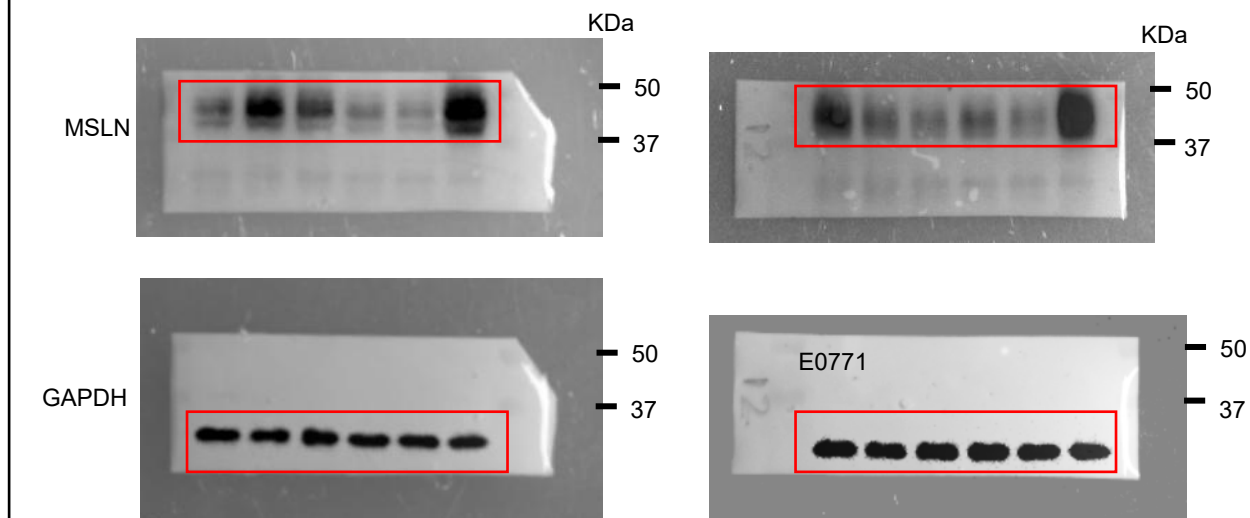

Fig S7B

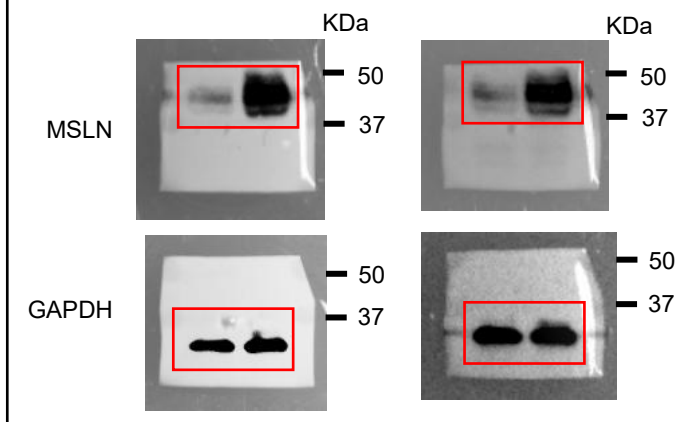

Fig S8D

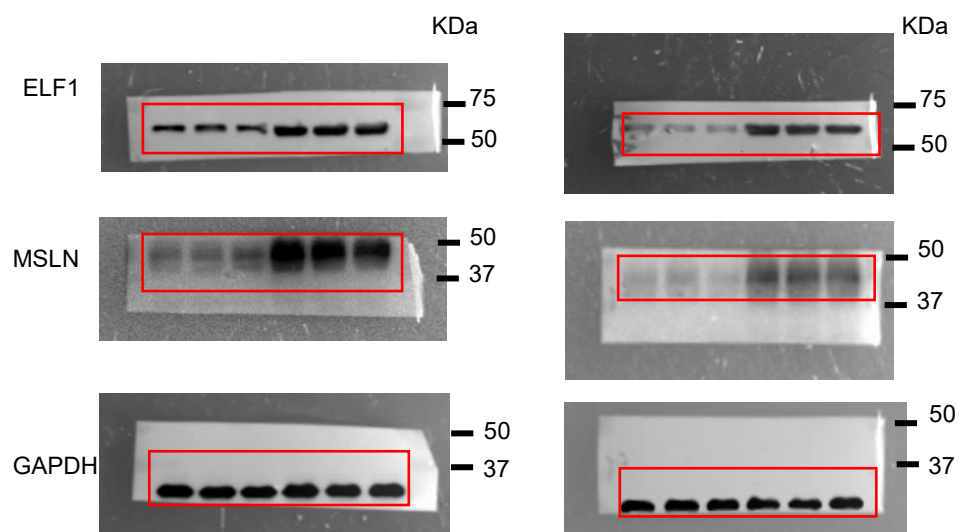

Fig S8E

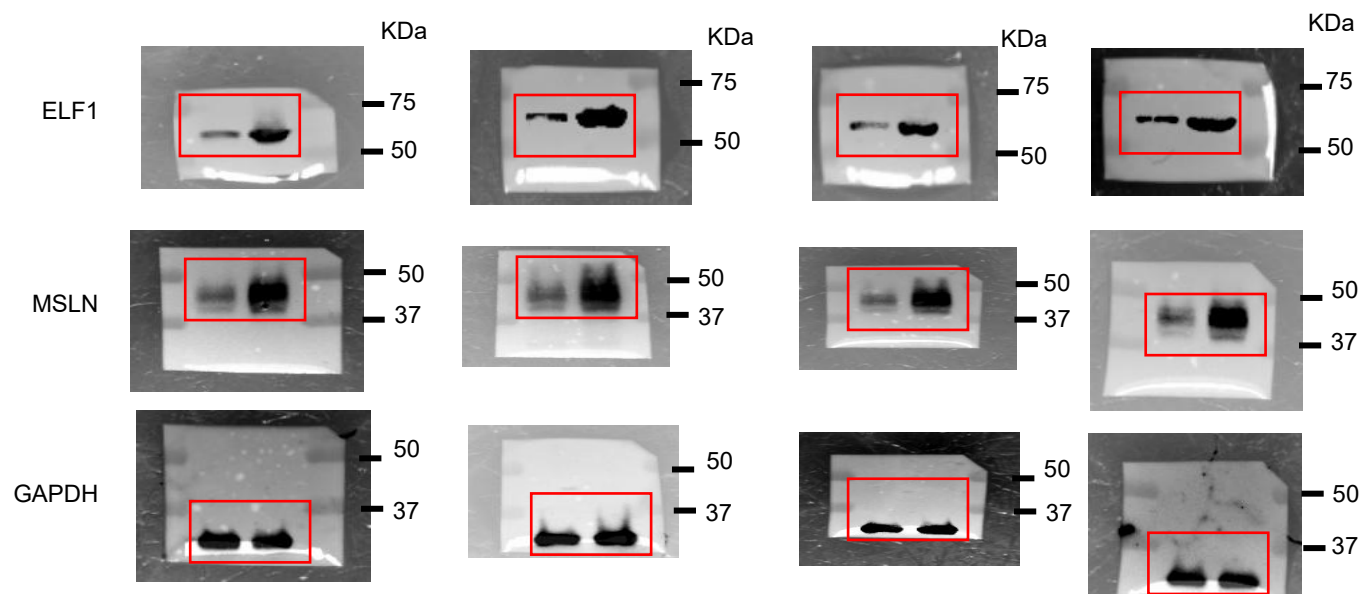

Fig S9A

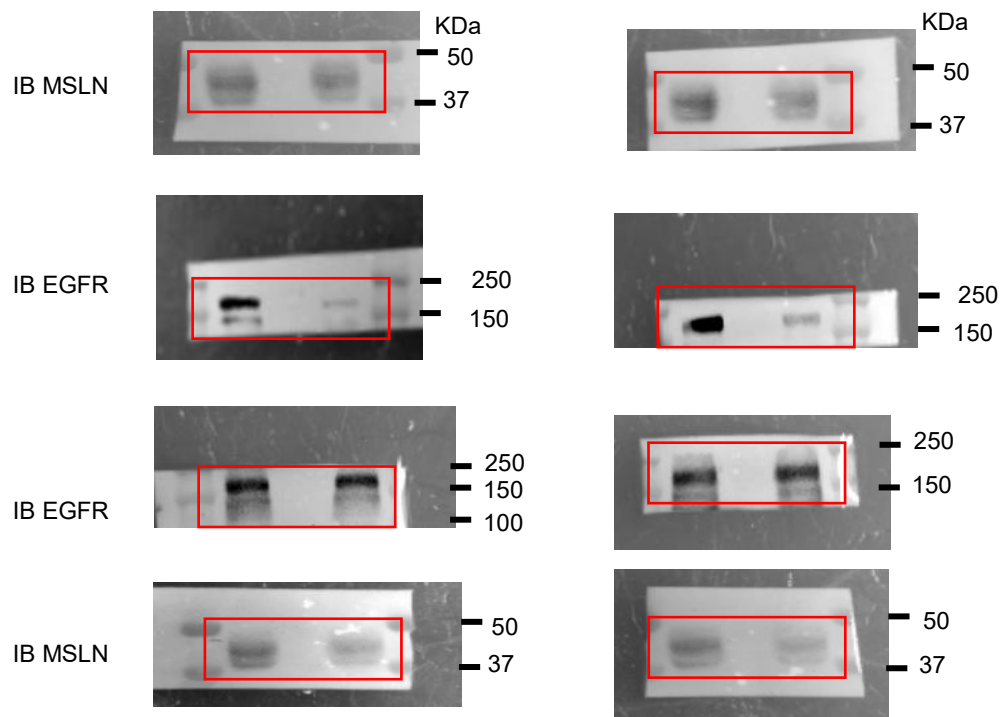

Fig S9D

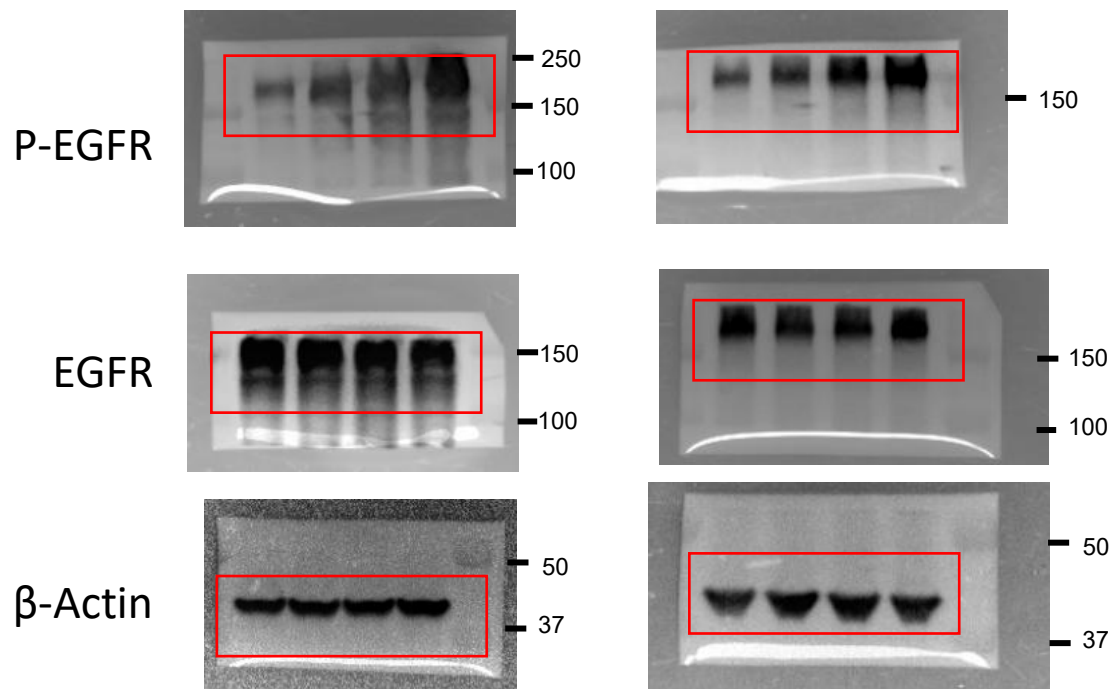

Fig S9E

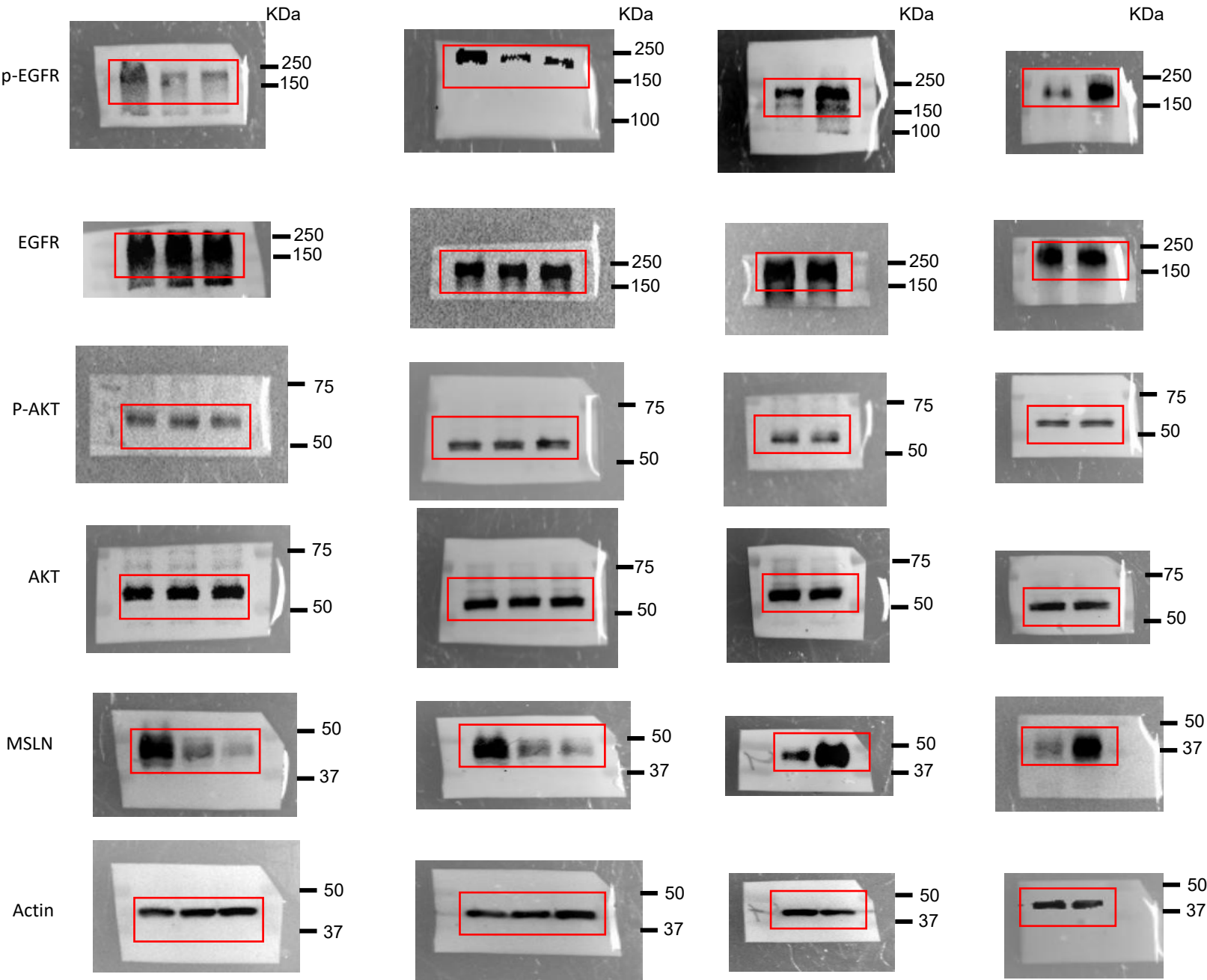

Fig S10B

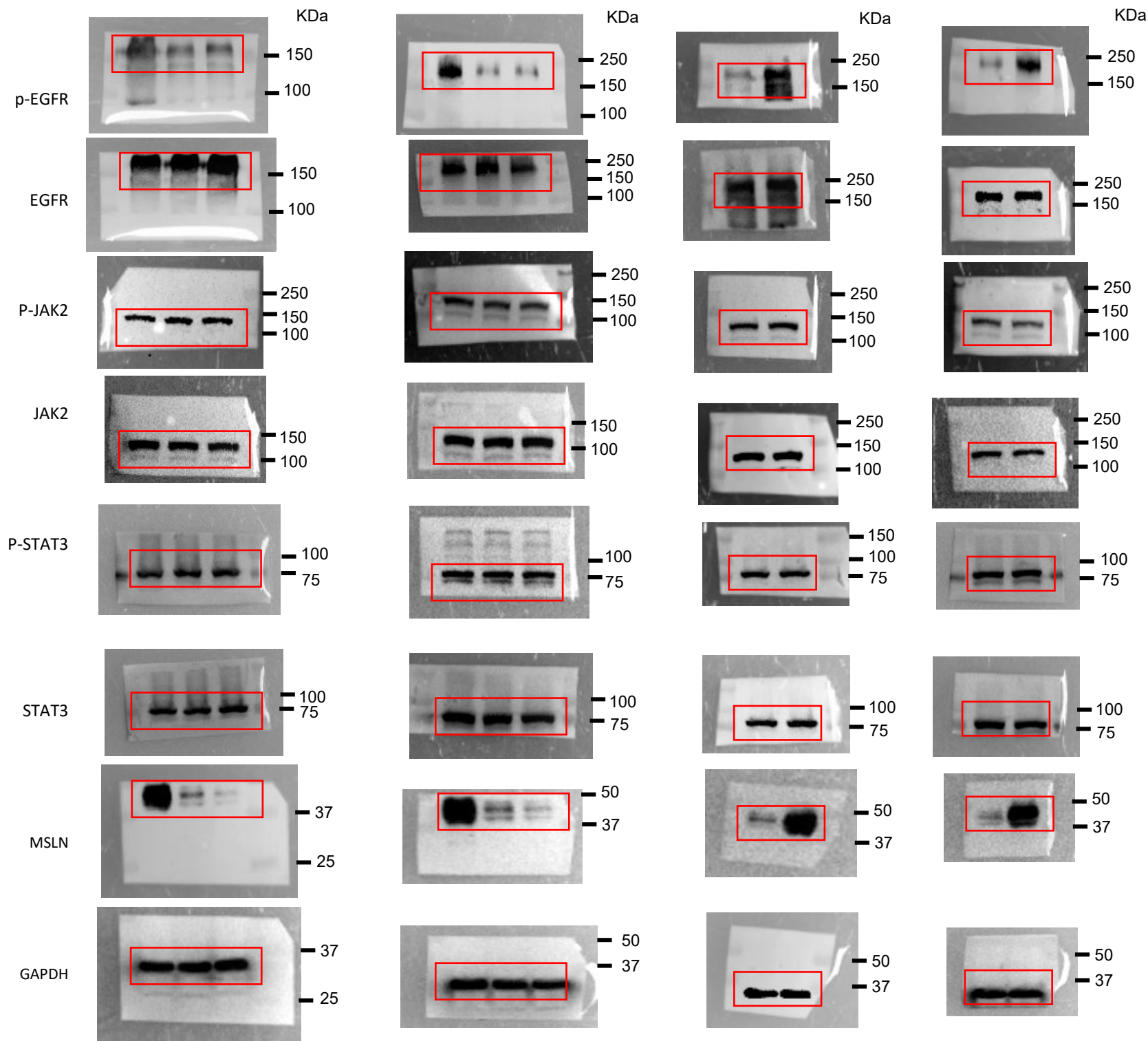

Supplement: Supplementary file 2 — Western Blots file [file 41420_2025_2835_MOESM2_ESM.pdf]
